# Comparative analysis of methods for detecting interacting loci

**DOI:** 10.1186/1471-2164-12-344

**Published:** 2011-07-05

**Authors:** Li Chen, Guoqiang Yu, Carl D Langefeld, David J Miller, Richard T Guy, Jayaram Raghuram, Xiguo Yuan, David M Herrington, Yue Wang

**Affiliations:** 1 Bradley Department of Electrical and Computer Engineering, Virginia Polytechnic Institute and State University, Arlington, VA, USA; 2 Department of Biostatistical Sciences, Wake Forest School of Medicine, Winston-Salem, NC, USA; 3 Department of Electrical Engineering, The Pennsylvania State University, University Park, PA, USA; 4 Department of Internal Medicine, Wake Forest School of Medicine, Winston-Salem, NC, USA

## Abstract

**Background:**

Interactions among genetic loci are believed to play an important role in disease risk. While many methods have been proposed for detecting such interactions, their relative performance remains largely unclear, mainly because different data sources, detection performance criteria, and experimental protocols were used in the papers introducing these methods and in subsequent studies. Moreover, there have been very few studies strictly focused on comparison of existing methods. Given the importance of detecting gene-gene and gene-environment interactions, a rigorous, comprehensive comparison of performance and limitations of available interaction detection methods is warranted.

**Results:**

We report a comparison of eight representative methods, of which seven were specifically designed to detect interactions among single nucleotide polymorphisms (SNPs), with the last a popular main-effect testing method used as a baseline for performance evaluation. The selected methods, multifactor dimensionality reduction (MDR), full interaction model (FIM), information gain (IG), Bayesian epistasis association mapping (BEAM), SNP harvester (SH), maximum entropy conditional probability modeling (MECPM), logistic regression with an interaction term (LRIT), and logistic regression (LR) were compared on a large number of simulated data sets, each, consistent with complex disease models, embedding *multiple *sets of interacting SNPs, under different interaction models. The assessment criteria included several relevant detection power measures, family-wise type I error rate, and computational complexity. There are several important results from this study. First, while some SNPs in interactions with strong effects are successfully detected, most of the methods miss many interacting SNPs at an acceptable rate of false positives. In this study, the best-performing method was MECPM. Second, the statistical significance assessment criteria, used by some of the methods to control the type I error rate, are quite conservative, thereby limiting their power and making it difficult to fairly compare them. Third, as expected, power varies for different models and as a function of penetrance, minor allele frequency, linkage disequilibrium and marginal effects. Fourth, the analytical relationships between power and these factors are derived, aiding in the interpretation of the study results. Fifth, for these methods the magnitude of the main effect influences the power of the tests. Sixth, most methods can detect some ground-truth SNPs but have modest power to detect the whole set of interacting SNPs.

**Conclusion:**

This comparison study provides new insights into the strengths and limitations of current methods for detecting interacting loci. This study, along with freely available simulation tools we provide, should help support development of improved methods. The simulation tools are available at: http://code.google.com/p/simulation-tool-bmc-ms9169818735220977/downloads/list.

## Background

Genome-wide association studies (GWAS) have been widely applied recently to identify SNPs associated with common human diseases [1-9], including cardiovascular diseases [6,10-13], diabetes [6,14-18], lupus [19-21], autoimmune diseases [22], autism [23], and cancer [24-27]. However, with few exceptions [13,15,17,24], the discovered genetic variants with significant main effects account for only a small fraction of clinically important phenotypic variations for many traits [5,28]. While there are multiple causes for missing some well-known genetic risk factors or disease heritability (including e.g., rare variants not genotyped in a GWAS study), a frequently cited reason is that most common diseases have complex mechanisms, involving multi-locus gene-gene and gene-environment interactions [5,28-31]. For detecting interacting loci in high dimensional GWAS data with sufficient power and computational feasibility, some pioneering work, with promising results, has been reported, encompassing: i) real GWAS study papers, as cited above; ii) interaction detection methodology [32-44]; iii) theoretical papers that characterize the principle problem (interaction detection) and its challenges [30,45-47]; iv) review and methods comparison papers [29,31,47-51].

### Novel Methods for Detecting Interacting SNPs

A variety of SNP interaction detection methods have been recently proposed. In particular, multifactor dimensionality reduction (MDR) [33] measures the association between SNPs and disease risk using prediction accuracy of selected multifactor models. Full interaction model (FIM) [41] applies logistic regression, 3 using*^d^*-1 binary variables constructed based on a *d*-SNP subset. Information gain (IG) [34,52] measures mutual information to assess multi-locus joint effects. Bayesian epistasis association mapping (BEAM) [32] treats the disease-associated markers and their interactions via a Bayesian partitioning model and computes, via Markov chain Monte Carlo (MCMC), the posterior probability that each SNP set is associated with the disease. SNP harvester (SH) [39] proposes a heuristic search to reduce computational complexity and detect SNP interactions with weak marginal effects. Random forest (RF) [44] is an ensemble classifier consisting of many decision trees, each tree using only a subset of the available features for class decision making. Thus, the detected features (SNPs) are the ones most frequently used by trees in the ensemble. Logic regression (LOR) [36] identifies interactions as Boolean (logical) combinations of SNPs. In [42], an extension of logic regression was also proposed to identify SNP interactions explanatory for the disease status, with two measures devised for quantifying the importance of these interactions for the accuracy of disease prediction. Treating SNPs and their interaction terms as predictors, penalized logistic regression (PLR) [37] maximizes the model log-likelihood subject to an L2-norm constraint on the coefficients. Related to FIM and PLR, adaptive group lasso (AGL) [43] adds all possible interaction effects at first and second order to a group lasso model, with L1-norm penalized logistic regression used to identify a sparse set of marginal and interaction terms. Maximum entropy conditional probability modeling (MECPM) [40], applying a novel, deterministic model structure search, builds multiple, variable-order interactions into a phenotype-posterior model, and is coupled with the Bayesian Information Criterion (BIC) to estimate the number of interaction models present. Logistic regression with an interaction term (LRIT) has been widely applied to detect interactions [35]. It treats the multiplicative term between SNPs, along with individual SNP terms, as predictors in the logistic regression model.

### Evaluation of Methods to Detect Interacting SNPs

Despite strong current interest in this area and a number of recent review articles [29,31,47-51], no commonly accepted performance standards for evaluating methods to detect multi-locus interactions have been established. For example, one might choose to evaluate power to detect individual SNPs involved in interactions, or power to precisely detect whole (multi-SNP) interactions. Moreover, the relationship between the power to detect interacting loci and the factors on which it depends (penetrance, minor allele frequency (MAF), main effects, and LD), while considered in some previous studies [32,41,43,45,53], has not been fully investigated, either experimentally or analytically. Most importantly, although some assessment and performance comparison was undertaken both in the original papers proposing new methods [32-34,39,41,43] and in the comparison papers [49,50], it is difficult to draw definitive conclusions about the absolute and relative performance of these methods from this body of studies due to the following: (1) each study was based on a different simulation data set and a different set of experimental protocols (including the detection power definition used, the sample size, the number of evaluated SNPs, and the computational allowance of methods). While use of different data sets and protocols may be well-warranted, as it may allow a study to focus on unique scenarios/application contexts not considered previously, it also makes it difficult to compare the performance of methods, excepting those head-to-head evaluated in the same study. Some methods were found to perform quite favorably in one study but poorly in others. For example, MDR [33] performed well in the original simulation study and the comparison paper [50], but poorly in subsequent studies [32,40,43]; (2) often, only simple cases were tested, which may not reflect the realistic application of a method. For example, a common practice is to include only a *single *interaction model in the data [32-34,39,41,50], whereas common diseases are usually complex, with multiple genetic causes [28], suggesting that *multiple *interaction models should be present. Our previous papers [40,54] considered multiple interaction models, but an insufficient number of data set replications to draw definitive conclusions on relative performance of methods [50]. also evaluated multiple interaction models, but only compared three methods, evaluated only one interaction power definition, and did not comprehensively evaluate the effects of penetrance, MAF, main effects, and linkage disequilibrium (LD) on power; (3) only limited interaction patterns were considered, e.g. 2-way interactions but no *higher-order *interactions in [43,49]. This is an important limitation, especially considering that data sets with 1000 or fewer SNPs were evaluated in these studies - in such cases, exhaustive evaluation of candidate pairwise interactions is computationally feasible, whereas *heuristic *search, which will affect detection power in practice, is necessitated if either higher order interactions or much larger SNP sets are considered. Thus, to more realistically assess detection power, either higher order interactions and/or more SNPs should be considered; (4) Perhaps most critically, methods providing P-value assessments [32,39,41] evaluated power for a given significance threshold, but did not rigorously evaluate the *accuracy *of the P-value assessment, i.e. whether the Bonferroni-corrected P-value *truly *reflects the family-wise type I error rate [55]. This evaluation is of great importance for methods that use asymptotic statistics [32,39,41], since it reveals whether or not the asymptotic P-value is a reliable detection criterion. Specifically, the P-value could be too liberal (in which case, more family-wise errors than expected will occur in practice and the estimated detection power is too optimistic) or too conservative (in which case the detection power estimate is too pessimistic). By not performing such assessment, it is unclear even whether use of P-values is providing a fair comparison of detection power between methods (*i.e*., for the same family-wise error rate) in [32,39,41]. We further note that although there were efforts to measure the type I error rate in [32,43,50], the evaluations were not based on the commonly used *family-wise error rate*, but rather on another definition of type 1 error [32] that does not directly reflect the Bonferroni-corrected P-value; (5) In most past studies [32-34,39,41,43,50], only a single definition of an interaction detection event (and, thus, a single measure of detection power) was used. However, this does not capture the full range of relevant detection events for some applications of GWAS. In particular, in some works an exact joint detection event is defined, *i.e*. detection is successful only if all SNPs involved in the interaction (and *only *these SNPs) are jointly detected [43,50]. This is a stringent definition that gives no credit to a method that detects a subset of the interacting SNPs (*e.g*. 3 of the SNPs in a 5-way interaction), even though such partial detection is clearly helpful if *e.g*. one is seeking to identify a gene pathway, or if the remaining SNPs in the interaction can be subsequently detected by applying more sensitive (and computationally heavy) methods. Exact detection is especially stringent when there are *multiple *interactions present, with the disease risk effectively divided between the multiple models. Finally, we note that individual methods have their own inductive biases and, thus, may perform better under different detection criteria - one method may find more ground-truth SNPs, while another may be more successful at finding whole interactions. Use of multiple power definitions can reveal these differences between methods; (6) Most of the proposed methods (*e.g*. MDR, FIM, BEAM, MECPM, SH) are designed to detect both main effects and interaction effects, while to date they have only been evaluated on data sets containing interactions. It is thus also meaningful to measure how effective they are at detecting SNPs with only main effects, and how many false positive *interactions *they detect involving main effect SNPs.

Finally, we note that there are very few true (strict) *comparison *papers - most studies have focused on developing new methods, with experimental evaluation not the central paper focus. Two exceptions are [50] and [49]. However, they both embedded only a single interaction model in the data and considered data sets with only 100 SNPs; Moreover, [50] evaluated only 2-way and 3-way interaction detection, while [49] evaluated only two-way interaction detection.

The aforementioned limitations of previous studies are not surprising because of the following challenges associated with comparison studies: (1) it is impractical to evaluate methods on all of the (numerous possible) interaction models; (2) multiple aforementioned factors (MAF, penetrance, LD) jointly decide interaction effects, which thus entails extensive study design, experimentation, and computational efforts; (3) many replicated data sets are required to accurately estimate power and family-wise type I error rate, further increasing computational burden; (4) computational costs of some methods are inherently high; thus a thorough evaluation of these methods is a difficult hurdle; and (5) fair evaluation criteria are not easily designed because distinct methods have different inductive biases and produce different forms of output (e.g., some give P-value assessments while others only provide SNP rankings); (6) there is no consensus definition of power when seeking to identify *multiple sets *of predictors that are jointly associated with outcomes of interest.

Addressing the above challenges, a ground-truth based comparative study is reported in this paper. The goals are three-fold: (1) to describe and make publicly available simulation tools for evaluating performance of any technique designed to detect interactions among genetic variants in case-control studies; (2) to use these tools to compare performance of eight popular SNP detection methods; (3) to develop analytical relationships between power to detect interacting SNPs and the factors on which it depends (penetrance, MAF, main effects, LD), which support and help explain the experimental results.

Our simulation tools allow users to vary the parameters that impact performance, including interaction pattern, MAF, penetrance (which together determine the strength of the association) and the sporadic disease rate, while maintaining the normally occurring linkage disequilibrium structure. Also, the simulation tools allow users to embed multiple interaction models within each data set. These tools can be used to produce any number of test sets composed of user specified numbers of subjects and SNPs.

**Figure 1:**
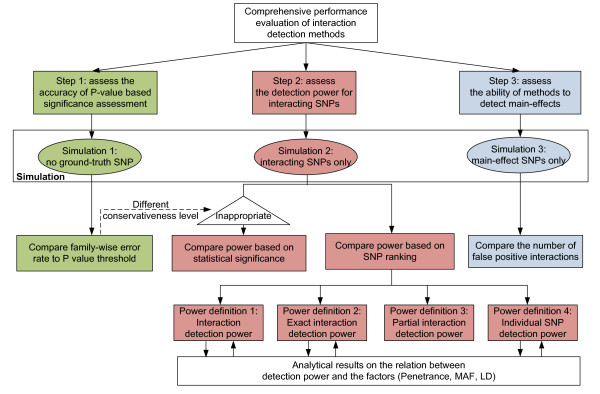
**A flowchart for the performance evaluation of interaction detection methods**.

Our comparison study, based on these simulation tools, involves thousands of data sets and consists of three steps, as graphically illustrated in Figure 1. Step 1 (with no ground-truth SNPs present) measures the empirical family-wise type I error rate, which has not been evaluated in many previous studies, and yet is critically important if the (*e.g*. P-value based) significance threshold is used as the criterion for detecting interacting SNPs.

In particular, foreshadowing our Step 1 results, we will find that *most *methods (except LR) in this study that produce P-values in fact produce conservative ones, with the degree of conservativeness method-dependent. Thus, using the same P-value threshold for all methods will not ensure the methods are being fairly compared, at a common family-wise error rate. Both for this reason, and because some of the methods do not even produce P-values, in Step 2 we evaluate detection power as a function of the number of top-ranked SNPs, rather than for a specified P-value threshold. Accordingly, note the logical structure in Figure 1, with the Step 1 results helping us to determine how to evaluate detection power in Step 2.

As aforementioned, Step 2 (with a variety of ground-truth interaction models present) investigates power. We formulate a more challenging, yet more realistic situation than most previous studies by including *multiple *ground-truth interaction models in each simulated data set. These simulations are motivated in part by our experience with complex genetic diseases such as autoimmune diseases, diabetes and end-stage renal disease [18,19,56,57]. In total, ninety different interaction models are investigated in this study, jointly determined by 5 underlying interaction types and 3 parameters, controlling penetrance, MAF, and LD. Step 3 investigates the power to detect main effect SNPs, *i.e*. we investigate how the methods (many of which are designed to detect both interactions and main effect SNPs) perform when only main effects are present in the data.

The main contributions and novelty of our comparison study are: (1) comprehensive comparison of state-of-the-art techniques on realistic simulated data sets, each of which includes multiple interaction models; (2) new proposed power criteria, well-matched to distinct GWAS applications (*e.g*., detection of "at least one SNP in an interaction"); (3) evaluation of the *accuracy *of (P-value based) significance assessments made by the detection methods; (4) investigation of detection of models with variable order interactions (up to 5th order) in SNP data sets; (5) new analytical results on the relationship between interaction parameters and statistical power; (6) investigation of the flexibility of interaction-detection methods, *i.e*. whether (and with what accuracy) they can detect both interactions and main effects; (7) discoveries concerning relative performance of methods (*e.g*., comparative evaluation of the promising recent method, MECPM). Since we are presenting a diversity of results, both experimental and analytical, to assist the reader in navigating our work, Figure 1 gives a graphical summary of our experimental steps, the results produced there from, and the connections between the different results, both experimental and analytical.

## Results

### Experimental Design and Protocol

We selected eight representative methods for evaluation, based on their reported effectiveness and computational efficiency. Seven of them (MDR, FIM, IG, BEAM, SH, MECPM and LRIT) are designed to detect interacting loci, with the remaining one based on the widely-used logistic regression model (LR). LR, using only main effect terms, serves as a baseline method to compare against all the interaction-detection methods, i.e., to see whether they give any advantage over pure "main effect" methods when the goal is simply to detect the subset of SNPs that either individually, or via interactions, are predictive of the phenotype. The description of the eight methods is given in the "Methods" part.

#### Simulation Data Sets

**Figure 2:**
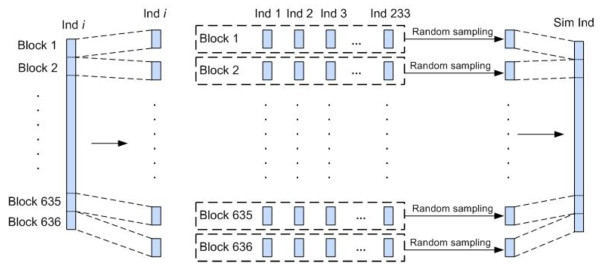
**A visual illustration of SNP "blocking" and random sampling, used for generating simulated individuals**. "Ind *i*" denotes the *i*th real individual, and "Sim Ind" denotes the simulated individual. First, genomes of the real individuals are segmented into a number of blocks; second, for each block, a genome segment is randomly drawn from the set of real individuals; finally, the randomly drawn genome segments, for all blocks, are stitched together to form a simulated individual.

Each data set contains individuals simulated from the control subjects genotyped by the 317K-SNP Illumina HumanHap300 BeadChip as part of the New York City Cancer Control Project (NYCCCP). To facilitate this investigation [40], a flexible simulation program was written that generates user defined sample size, number of SNPs, no missing data or missing data patterns consistent with the observed missing data in the original genome scan, and affected or unaffected disease status under the null hypothesis (i.e., no associations in the genome) or under the alternative hypothesis (i.e., hard-coded penetrance functions). Missing data is filled in completely at random and proportional to the allele frequencies in the original data. The data sets were produced as follows. Consider a matrix with 223 rows corresponding to NYCCCP individuals and 317,503 columns corresponding to the 317,503 SNPs. The elements of this matrix are the individual genotypes. The columns were partitioned into blocks of 500 SNPs, i.e. 636 blocks, with the last block containing only 3 SNPs. The simulated genome scan data for each individual was obtained by random draws (with replacement) from a real data matrix of 223 individuals and 636 blocks of 500 SNPs. Specifically, the simulated data for an individual was generated by randomly selecting the first block from the 223 individuals (rows), randomly selecting with replacement the second block from the 223 individuals, randomly selecting with replacement the third block from the 223 individuals, and so on. Thus the data retains the basic patterns of linkage disequilibrium (broken by strong recombination hotspots), missing data, and allele frequencies observed in the original genome scan data. The exception to this is only at the 635 breaks in the genome corresponding to the block boundaries. Figure 2 visually illustrates this simulation approach for randomly resampling genome scan data starting from the real NYCCP scans. The simulations presented here correspond to approximately 2000 subjects simulated under the alternative hypothesis described below and no missing data. Only autosomal loci are considered in the data.

The eight methods were applied to sets of 1000~10,000 SNPs selected at random from the autosomal loci. This number of SNPs is consistent with a GWAS study following an initial SNP screening stage and also with pathway-based association studies. When selecting SNPs, we first removed those with genotypes that significantly deviate from Hardy-Weinberg equilibrium, and then selected the desired number of ground-truth and "null" SNPs. For each replication data set, ground-truth SNPs were randomly selected, according to the requirements of MAF (within a narrow window of tolerance), and "null" SNPs were chosen completely at random. The simulations reported assume that the disease risk is explained by several ground-truth interaction models and the sporadic disease rate *S*, which accounts for the missing heritability and other disease-related factors. Let *P_r_*(*d_i_*), *r *= 1,2,...,*R *be the disease probability generated by *R *interaction models for the *ith *subject. Assuming all disease factors act independently, disease risk of this subject is then(1)

The simulation data sets have different ground truth interaction models *P_r_*(*d_i_*), *r *= 1,2,...*R *and the sporadic disease rate *S *for different steps. For Step 1, we did not embed any ground truth SNPs in the data sets; for Step 2, we embedded five interaction models in each data set; and for Step 3, we embedded five main-effect-only SNPs in each data set. In all three steps, we adjusted the sporadic rate *S *so that each data set has approximately 1,000 cases and 1,000 controls, the typical situation (balanced cases and controls) in GWAS studies, e.g. in Step 1, *S *= 0.5. The ground truth interaction models in Step 2 and the ground truth main-effect-only SNPs will be described later.

**Figure 3:**
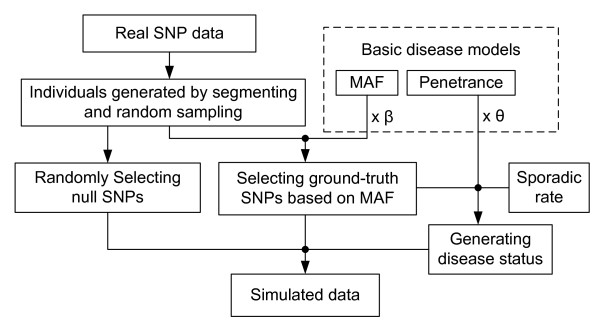
**A flowchart detailing all of the steps used in producing the simulated GWAS data sets**.

In Figure 3, we provide a flowchart detailing all of the steps (as described above) used in producing our simulated GWAS data sets.

The simulation approach used in this comparison study is the same as that used in [40]. Our simulation approach has one commonality with, but two main differences from the simulation approaches used in the previous methods and comparison study papers evaluating MDR, IG, FIM, SH, and BEAM [32-34,39,41,50]. Both in these papers and in our current study, all SNPs are consistent with Hardy-Weinberg Equilibrium. However, in these previous papers, the simulated data were purely synthetic, generated according to user-specified allele frequencies [29-31,36,38,47]. By contrast, our simulated data is obtained by resampling from real genome scan data and is thus more realistic, preserving the allele frequencies and LD structure manifested by the original genome scan data. Another resampling simulation method was proposed in [58,59], but this approach has not been used for evaluating the MDR, IG, FIM, SH, and BEAM methods. Another important distinction between our simulation method and other simulation methods lies in the phenotype generation. In our simulation, multiple interactions simultaneously exist in each data set (which is reasonable considering complex disease mechanisms) and jointly decide the phenotype; by contrast, other simulation methods usually embed only one SNP interaction (i.e., single interaction model) in each data set [32-34,39,41,50]. Also, we consider interactions with interaction order from 2 to 5, while most other simulations [33,34,39,41,50] only consider interactions with interaction order up to 3.

As mentioned previously, our simulation study consists of three main experimental steps, which we next more fully describe.

#### Step 1: assess family-wise type I error rate

An accurate family-wise type I error rate is crucial for methods that select candidate SNPs based on their P-values and for reliably comparing methods. If the family-wise type I error rate is either conservative or liberal, the P-value loses its intended meaning and does not reflect the actual false positive rate. That is, we will not be able to control how many false positives are detected by setting a (*e.g*. P-value based) threshold. For example, a method with a lower family-wise type I error rate than expected (based on the estimated P-value) sets a threshold that overestimates the empirical false positive rate; thus, fewer false positives (than the target) will be selected, likely also leading to fewer true associations being identified.

BEAM, SH and FIM detect significant SNPs based on P-values calculated from asymptotic distributions and heuristic searches. Thus, based on the preceding discussion, evaluating the accuracy of their P-value assessments is not only of theoretical importance (how well their proposed asymptotic distributions approximate the real distribution), but also of great practical necessity in applying these methods.

To evaluate the accuracy of P-value assessment, we replicated 1,000 data sets by repeatedly randomly selecting 1,000 null SNPs from the SNP pool, i.e. to easily assess family-wise type I error rate, no ground-truth SNPs were embedded in these data sets.

#### Step 2: assess power

In step 2, each data set has *N *SNPs, with 15 ground-truth SNPs and *N*-15 null SNPs, selected via the procedure described in the "*Simulation Data Sets*" subsection. *N *is chosen to be either 1000 or 10,000 for different experiments. There are several points to make regarding the number of SNPs we consider. First, assuming approximately 1000~10,000 SNPs is realistic for candidate gene and biological pathway studies where interaction detection is needed. Second, considering GWAS studies, a 0.15%~1.5% percentage of ground-truth SNPs realistically models the output of first stage SNP screening/filtering (which greatly reduces the number of candidate SNPs) in the widely-applied 2-stage GWAS detection process. Finally, the 1000~10,000 SNPs considered here is much larger than the 100 SNPs in the previous comparison study [49,50] and comparable to that considered in several other recent papers.

The 15 ground-truth SNPs each participate in one of 5 ground-truth SNP interactions, which contribute independently to the disease, as described by equation (1). There are three standard factors that determine interactions: penetrance, MAF and LD [3,7]. Penetrance is the proportion of individuals with a specific genotype who manifest the phenotype. For example, if all individuals with a specific disease genotype show the disease phenotype, then the penetrance value is 1 and the genotype is said to be "completely penetrant"; otherwise, it is "incompletely penetrant" [3]. LD is the non-random association of alleles of different linked polymorphisms in a population [7]. MAF is the frequency of the least common allele of a polymorphic locus. It has a value that lies between 0 and 0.5, and can vary between populations [7]. The 5 ground-truth SNP interactions are jointly determined by 5 basic model types and 3 (discrete-valued) parameters, controlling the MAF, penetrance, and LD, which will be specified later. Based on the choices for these 3 parameters, there are 3 × 3 × 2 = 18 possible parameter configurations (so the aforementioned ninety models are generated by the 5 basic model types, each with 18 different parameter settings). Each configuration is applied simultaneously to the 5 basic models, thus yielding 5 fully specified interaction models for a given data set. With some allowable randomness in the 5 new interaction models, we generated 100 replication data sets for each configuration with *N = *1000, and 10 replication data sets for one typical configuration with *N = *10,000; thus we have in total 18 × 100+10 = 1,810 data sets in step 2, involving 18 × 5 = 90 interaction models.

**The 5 basic models **vary in interaction order, genetic models (dominant, recessive, or additive), incomplete/complete penetrance, MAF, and marginal effects. To indicate the strength of interaction effects and main effects for each basic model, we calculated the odds ratio by dichotomizing the genotypes of each interaction into a group with the lowest penetrance value (usually with "0" penetrance) and another group with higher penetrance values (the specific calculation can be found in section S4 of the Additional file 1).

The 5 basic models are defined by the penetrance tables and MAFs below. The penetrance function is the probability of disease given the individual's genotype. Thus, the penetrance tables show the probability of developing disease given the genotypes [3,60], with each table entry being the disease probability conditional on the specific single or multi-locus genotypes. The interaction models are motivated by our experience studying complex genetic traits where there are multiple loci contributing to disease risk. Specifically, the simulation study is motivated by our experience in autoimmune diseases, diabetes and renal diseases where there are some larger effects (e.g., human leukocyte antigen region in autoimmune diseases such as systemic lupus erythematosus, neonatal lupus, and juvenile arthritis [19]; and gene APOL1 in end-stage renal disease in African Americans [18]), and multiple modest to smaller effects with 1.1 < odds ratios < 1.3. To date, there are few robustly established (i.e., with convincing discovery evidence on multiple replications in independent cohorts) gene-gene interactions in the human disease literature. Thus, we attempted to be consistent with the complex genetic disease paradigm and assumed multiple loci, several interacting, contribute to the risk of disease. We examined combinations of SNPs in the lupus genome-wide scan (Harley et al, 2008) to estimate some examples of potential two-locus interactions as well as constructed other higher-order interactions consistent with traditional interpretations of Mendelian inheritance (i.e., dominant, additive or recessive genetic model) but spanning multiple loci. Some interactions are based on a two-locus, common allele with a low penetrance model as might be hypothesized in diabetes from the "thrifty gene hypothesis" [56] and other multi-locus models are modest penetrance models for the low frequency alleles. Additional motivation comes from studies of epistasis [57]. The five locus interaction is a conjectural one that should challenge these analytic methods.

**Basic model 1**-.two-locus interaction under a dominant model for the major allele. The model is for two very common but low penetrant alleles. The MAFs at these two loci are both 0.25. This model is expected to generate 62 cases per 1000 subjects. The odds ratio is 1.16 for the joint interaction effect between *A *and *B*, and 1.15 for main effects of both *A *and *B*. This model simulates the situation of common disease where the major allele is disease-related but with weak interaction effects. "M1" denotes model 1.  denotes the homozygous major allele genotype of SNP A;  denotes the heterozygous genotype of SNP A;  denotes the homozygous minor allele genotype of SNP A; likewise for the notations in the other basic models.

**Basic model 2**- two-locus interaction for common alleles under a dominant genetic model at each locus. The minor allele frequencies are 0.20 for locus *A *and 0.30 for locus *B*. This model is expected to generate 102 cases per 1000 subjects. The odds ratio is 3.79 for the joint interaction effect between *A *and *B*, 1.89 for the main effect of *A *and 1.56 for the main effect of *B*. This model simulates the situation that the minor allele is disease-related, and both interaction effects and main effects are strong.

**Basic model 3**- three-locus interaction, common alleles, incomplete penetrance. The MAFs at the three loci are 0.40 for *A*, 0.25 for *B*, and 0.25 for *C*. This model is expected to generate 46 cases per 1000 subjects. The odds ratio is 2.28 for the joint interaction effect among *A, B *and *C*, 1.16 for the main effect of *A*, 1.25 for the main effect of *B*, and 1.25 for the main effect of *C*.

**Basic model 4**- three-locus interaction among common alleles. The minor allele frequencies are 0.25 for *A*, 0.20 for *B*, and 0.20 for *C*. This model is expected to generate 26 cases per 1000 subjects. The odds ratio is 5.79 for the joint interaction effect among *A, B *and *C*, 2.45 for the main effect of *A*, 1.06 for the main effect of *B*, and 1.06 for the main effect of *C*. This model has strong interaction effects and a strong main effect at *A*, but weak main effects at *B *and *C*. Two-SNP subsets of the three-locus interaction, {*A, B*} and {*A, C*}, also have strong effects.

**Basic model 5**- five-locus interaction among common alleles. It assumes a MAF of 0.30 at each locus and has a penetrance value of 0.63 if the minor allele is present at each locus; and 0 otherwise. In equation form, the penetrance function is:

where *D *means the subject gets disease. This model is expected to generate 22 cases per 1000 subjects. The odds ratio is 4.48 for the joint interaction effect among the five loci, and 1.09 for the main effect at all five loci. This model simulates the situation of significant high-order interaction effects but weak main effects.

**Three parameters **are used to assess the robustness of the various methods to variations in penetrance, MAF, and LD, because i) as aforementioned, penetrance, MAF, and LD jointly define the disease model, and thus decide the disease status; ii) it is of interests, in the field of SNP interaction detection, to explore how detection power varies with these parameters [32,34,41,43,50]; iii) we have derived the analytical relationships between interaction effects and these parameters in the Additional file 1, so a simulation study using these parameters provides us the opportunity to validate the analytical relationships in an empirical way. For each basic model, we control its penetrance by multiplying every value in the penetrance table by the penetrance factor (multiplier) *θ *∈{1,1.3,1.4} (the larger *θ *is, the larger disease risk there will be); we discount the MAF by multiplying the MAF of each SNP by a MAF factor *β *∈{1,0.9,0.7} (the larger *β *is, the larger frequency the minor allele will have); and to control the LD level, we replace each ground-truth SNP by an "LD SNP", which has a certain correlation coefficient *l *∈{0.8,*null*} with the ground-truth SNP (*l *= *null *means we do not replace the ground-truth SNP). The "LD SNP" simulates the realistic case where the ground-truth SNP is not directly genotyped; in this case we may detect a SNP in LD with the ground-truth SNP. For example, for basic model 2, under parameters *θ, β, l*, the MAFs are 0.2 * *β *for locus *A *and 0.3 * *β *for locus *B, θ *determines a new penetrance function shown below, and if *l *= 0.8, we replace *A*/*B *by a SNP correlated to *A*/*B *with correlation coefficient 0.8.

The theoretical, analytical relationship among penetrance, MAF, and statistical significance of an interaction model is investigated in the Additional file 1, with these results also summarized in the "Experimental Results" section.

#### Step 3: assess the power to detect SNPs with only main effects

Most of the interaction-detection methods are designed to find either interactions or main effects (*e.g*. MDR, FIM, BEAM, MECPM and SH). Thus, it is meaningful to see how these methods fare in detecting main effects and also whether they detect false positive interactions (which may involve either null and/or main effect SNPs) when there are *only *main effects present.

In Step 3, we simulated 100 replication data sets, following a similar approach

as in Step 2. Each data set includes five main-effect ground truth SNPs and 995 null SNPs. The penetrances and MAFs for the five ground truth SNPs are:

**SNP 1. **Dominant model for the major allele, low penetrance, MAF = 0.25.

**SNP 2. **Additive model for the minor allele, MAF = 0.3.

**SNP 3. **Additive model for the minor allele, MAF = 0.4.

**SNP 4. **Recessive model for the minor allele, high penetrance, MAF = 0.25.

**SNP 5. **Dominant model for the minor allele, low penetrance, MAF = 0.3.

Although SNP 1 and SNP 5 have relatively weaker effects, we still included them because (1) they also affect many subjects' disease status, since a large proportion of subjects carry the disease genotype of SNP1 and SNP 5 (which simulates common-disease markers); (2) our experimental results will show that these weak-effect SNPs differentiate the performance of the methods.

Note that we configured the methods to detect both main effects *and *interaction effects since, in practice, it will not be known whether interactions are present or not.

### Design of Performance Measures

The performance of the methods is evaluated by the accuracy of P-value assessment, various definitions of power, reproducibility, and computational complexity.

#### A. Family-wise type I error rate (the accuracy of P-value assessment)

There are 1,000 SNPs in each data set. Thus there are multiple comparison effects, and the P-values obtained by the methods are accordingly adjusted by Bonferroni correction. In this way, the accuracy of P-value assessment is represented by the family-wise type I error rate: an error event occurs on a data set with no ground truth SNPs if there are *any *(necessarily false) positive detections. Since SH, MDR and FIM use Bonferroni correction, we measure the accuracy of their P-value assessments by how well the significance threshold (P-value) agrees with the family-wise type I error rate.

#### B. Various power definitions and the ROC curve

Power can be defined in several ways, depending on what we desire to measure. We next give several power definitions experimentally evaluated in the sequel.

##### Power to progressively detect interactions (Power definition 1)

*the frequency with which a model's ground-truth SNPs are ranked within the top K positions*. Several comments are in order here. First, it is important to note that the significance threshold is not being applied to define power because (1) the methods' P-value assessments are, as noted earlier, conservative (as shown in the sequel), and (2) not all methods provides significance assessments (*e.g*. IG and MECPM). Second, in our experiments, the ranking of a SNP is decided by the strength of effect of the most significant interaction that includes this SNP. Third, note that each data set contains *multiple *interaction models, with the detection power measured separately for each model. In measuring the power to detect SNPs in a given interaction amongst the top *K *SNPs, we are only interested in whether the ground-truth SNPs in the interaction are ranked higher than null SNPs, not whether they are ranked higher than ground-truth SNPs from other interactions that are present. Accordingly, when measuring the power to detect SNPs in a given interaction, we do not rank ground-truth SNPs from other interactions, but only rank SNPs from the given interaction and all null SNPs. For an *M*-way interaction, let {*x_K_*(*i*), *i *= 1,2,...,100} be the number of its ground-truth SNPs reported within the top *K *SNPs over the 100 replicated data sets. The power for this interaction model is then given by:(2)

We can also define power over the *entire *ground-truth SNP set by setting *M *= 15 and considering all ground-truth SNPs in the ranking.

##### Power to precisely detect interactions (power definition 2: exact interaction power)

*for an M-way ground-truth interaction, how likely it is detected amongst the top K M-way candidates produced by a method*. This power definition evaluates the sensitivity to detect the interaction *as a whole*, rather than as individual SNPs. Again, similar to power *definition 1*, in evaluating the top *K M*-way candidates, we only consider *M*-way combinations that include ground-truth SNPs from the interaction of interest and null SNPs, *i.e*. we exclude *M*-way SNP combinations involving any SNPs that participate in other ground truth interactions. Mathematically, for an *M*-way interaction {*s*_1_,..., *s*_*M*_}, in the *ith *data set, if {*s*_1_,..., *s*_*M*_} is detected within the top *K M*-way candidates, *x*_2, *i*_(*K*) = 1; otherwise, *x*_2, *i*_(*K*) = 0. Power *definition 2* is then given by:

##### Power to detect at least 1 SNP in the ground-truth interaction (power definition 3: partial interaction power)

As revealed by the definitions of the interaction models, a *subset *of the interacting SNPs may have strong association to disease risk. Detecting an interaction subset should be acceptable since this gives a good "clue" to help further identify the complete interaction. We thus give power *definition 3 *as follows: for an *M*-way interaction model {*s*_1_,...,*s_M_*}, if any SNP from {*s*_1_,...,*s_M_*} is within the top *K *SNPs reported by the methods (excluding other ground-truth SNPs that do not participate in this interaction model), *x*_3,*i*_(*K*) = 1; otherwise, *x*_3,*i*_(*K*) = 0. Power *definition 3 *is then given by:

##### Power to detect individual SNPs (power definition 4: single SNP power)

The power definitions above ignore differences between SNPs within the same interaction, *e.g*., differences in MAF, asymmetric penetrance table and thus different main effects, which may largely affect their potential for being detected. So it is also necessary to see how well individual ground-truth SNPs with different MAFs, penetrances, and main effects, are detected by the 5 methods. Accordingly, we give power *definition 4 *as follows. For a ground-truth SNP *s_j_, j *= 1,2,...,15, if *s_j _*is within the top *K *SNPs reported (excluding the other ground-truth SNPs), *x_i_*(*K*) = 1; otherwise, *x_i_*(*K*) = 0. The single SNP power for *s_j _*is then given by:

##### ROC curve

We also evaluate the methods via the ROC curve, which shows how many ground-truth SNPs are detected for a given false positive SNP count.

#### C. Reproducibility

The estimated power, even if high, could deviate significantly across different data set replications, due to the inherent randomness in our simulation approach. Thus, we also want to see how reproducible the detection power is over the data set replications. To evaluate this, we measure the standard deviation of the estimated power across the replicated data sets.

#### D. Computational complexity

Computational complexity was measured by the execution time and memory occupancy of the methods for the same platform.

### Experimental Results

In Step 1, we evaluated the three methods with asymptotic statistics (FIM, BEAM and SH). In Step 2, we evaluated all eight methods (as described in the "Method" section) on the 1000-SNP data sets, and six methods (FIM, IG, BEAM, MECPM, SH and LR) on the 10,000-SNP data sets - we do not evaluate MDR for the 10,000-SNP data sets because the high memory occupancy of the MDR software prevents this evaluation. We also evaluated six methods (MDR, FIM, BEAM, MECPM, SH and LR) in Step 3 - we do not evaluate IG and LRIT, because, by design, they only output multi-locus interaction candidates, and thus are inappropriate to be assessed in Step 3's main effect evaluation. Specifically, IG and LRIT will necessarily have 0 true positives, no matter how well they detect interactions involving the main-effect-only SNPs, since in Step 3 only "singlet" main effects are considered to be true positives. MDR, BEAM, SH and MECPM were all implemented using the authors' freely available software. LR, LRIT, FIM and IG were implemented using C++, with the software freely available. The eight methods were tested on the same platform: OS: Windows, CPU: 3G, RAM: 2G. The parameters used by the respective methods follow their default settings wherever possible. We only modified one parameter when testing MDR: we used its heuristic search (1 hour execution time limit) instead of exhaustive search when testing MDR on the 1000-SNP data sets in step 2, because exhaustive search of MDR required huge memory and quite impractically high computational cost - when implementing MDR with exhaustive search, our machine crashed from running out memory; moreover, the *estimated *exhaustive-search MDR execution time for a 1000-SNP, 2000-sample data set is 1.4 × 10^6 ^seconds (roughly 15 years) on our platform. Here we compare the eight peer methods along several performance fronts. The results are then further evaluated and summarized in the "Discussion" section.

#### Accuracy of P-value assessment in step 1

Based on the definition in the subsection "Design of Performance Measures", we tested the accuracy of P-value assessment for BEAM, SH, and FIM on the 1,000 data sets in step 1. Regarding the other methods, IG and MECPM do not give significance assessments, while the significance assessment of MDR is (necessarily) accurate since it uses random permutation testing (However, it should also be noted that MDR only evaluates the significance of the *top-ranking *interaction. Thus, in practice, MDR does not in fact use a P-value to practically set an interaction detection threshold.). The average family-wise type I error rates at different significance thresholds were calculated. Since each interaction order has a different Bonferroni penalty, we separately list the results for 1st, 2nd, and 3rd orders, shown in Table 1. BEAM, SH and FIM all have accurate family-wise type I error rates at 1st order, but give conservative results (empirical family-wise type I error rate is less than expected) at 2nd and 3rd order. BEAM is the most conservative and FIM the least. Thus, the P-values generated by these methods are conservative, and not to the same degree. Thus the estimation of power (at the targeted type I error rate value) is likewise both conservative and not truly comparable across the methods. There are multiple causes for this conservativeness, which we subsequently discuss.

**Table 1:** The average family-wise type I error rates (step 1) for BEAM, SH and FIM under the significance threshold of 0.1 (after Bonferroni correction). More results can be found in the Additional file 1.

family-wise type I error rate	BEAM	SH	FIM
1st order	0.094	0.084	0.097

2nd order	0	0.026	0.032

3rd order	0	0.002	0.006

#### Power (definition 1) and ROC curve in step 2

**Figure 4:**
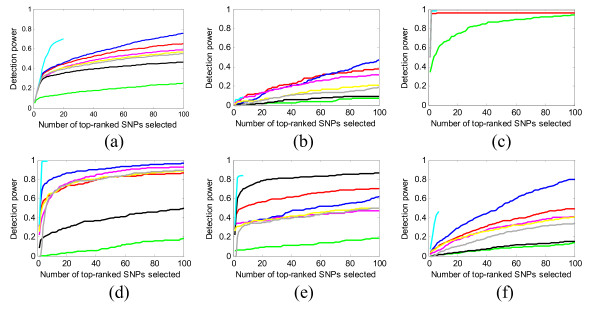
**Power evaluation (*definition 1*) of the eight methods on 100 replication data sets with parameter setting: *θ *= 1.4, *β *= 1, *l *= *null***. (a) evaluates the power on the whole ground-truth SNP set, and (b) (c) (d) (e) (f) evaluate the power individually on the 5 interaction models. Blue curve - SH, magenta curve - FIM, green curve - MDR, black curve - IG, cyan curve - MECPM, grey curve - LRIT, yellow curve - LR.

**Figure 5:**
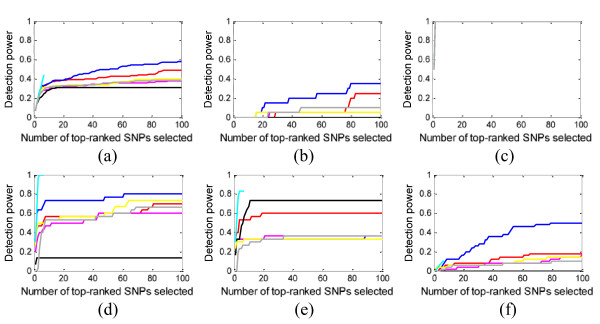
**Power evaluation (*definition 1*) of six methods on 10 replication data sets with parameter setting: *θ *= 1.4, *β *= 1, *l *= *null***. (a) evaluates the power on the whole ground-truth SNP set, and (b) (c) (d) (e) (f) evaluate the power individually on the 5 interaction models. In (c), all the methods have overlapped power curve at the upmost part of the figure. Magenta curve - FIM, black curve - IG, red curve - BEAM, blue curve - SH, cyan curve - MECPM, grey curve - LRIT, yellow curve - LR.

We measured power (*definition 1*) for each interaction model and also for the entire 15-SNP ground-truth set. Figure 4 and 5 show some of our results for 1000 SNPs and 10,000 SNPs in each data set, respectively. Many more results, under different parameter configurations, are given in the Additional file 1.

For the 1000-SNP case (Figure 4), although the methods can detect some SNPs with strong interacting effects (Figure 4(c), model 2), most of the methods (MDR, BEAM, IG, FIM, SH, and LR) miss many other ground-truth interacting SNPs at a low false positive SNP count (i.e., for small K) (Figure 4(b), (d), (e) and 4(f)); further increases in power are modest and are only attained by accepting many more false positive SNPs. Comparatively, MECPM performs quite well on most interaction models (including the difficult five-way (Figure 4(f)) and the three-way interactions (Figure 4(d) and 4(e)), except for model 1 (Figure 4(b)). Only a partial curve is shown for MECPM because MECPM uses the BIC criterion to choose its model order (and, thus, the number of interactions) [40]. Few true SNPs are added as *K *is increased beyond the BIC stopping point -- MECPM has high specificity at the BIC stopping point [40] (MECPM specificity = 0.99 for the whole ground truth SNP set at the BIC stopping point). Accordingly, MECPM execution was terminated shortly after the BIC stopping point. From Figure 4(a), MECPM is overall the best-performing method, with SH second, BEAM third, FIM fourth, the baseline LR fifth, LRIT sixth, IG seventh and MDR eighth. Individual methods perform more favorably for certain models, *e.g*. IG performs well for a 3-way model (Figure 4(e)). Also, all methods tend to detect more interacting SNPs with strong main effects than those with weak main effects (power of all the methods on models 2 and 3 is generally higher than on models 1, 4, and 5). We give some explanation for these results in the "Discussion" section.

For the 10,000-SNP case (Figure 5), we have similar observations as in the 1000-SNP case, except that the general performance of all methods is degraded. It is worth noting that all the methods perform comparably to their 1000-SNP detection power for model 2 (Figure 5(c)), and MECPM also performs comparably to its 1000-SNP detection power for models 3 and 4 (Figure 5(d) and 5(e)). MECPM is the overall best-performing method, with SH second, BEAM third, LR fourth, LRIT fifth, FIM sixth and IG seventh.

#### Impact of penetrance, MAF, and LD on power (definition 1)

**Figure 6:**
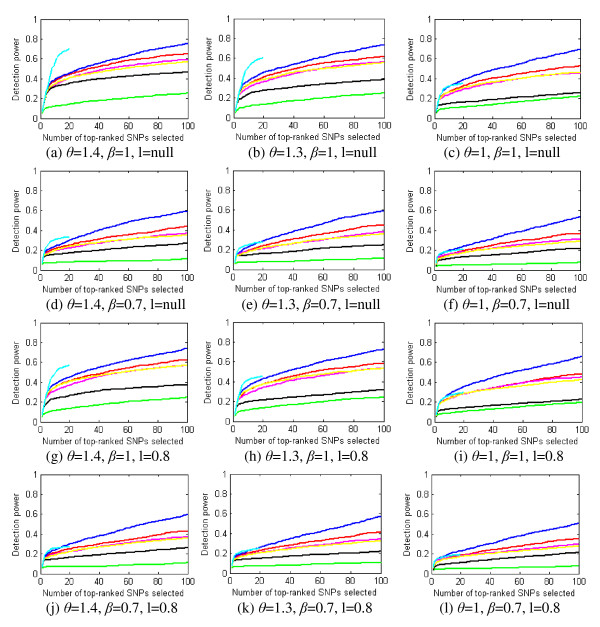
**The impact of penetrance value (*θ*), MAF (*β*), and LD factor (*l*) on power for the whole ground-truth SNP set**. Blue curve - SH, magenta curve - FIM, green curve - MDR, black curve - IG, cyan curve - MECPM, yellow curve LR..

Figure 6 shows the power for different penetrance, MAF, and LD factors. The power is calculated based on the whole ground-truth SNP set. More detailed results are given in the Additional file 1. From Figure 6, a smaller penetrance value or MAF significantly degrades the power curves of the methods. Among the methods, SH is most robust to changes in penetrance and MAF, and IG is most sensitive to these changes.

#### Reproducibility of power (definition 1)

We measured the reproducibility by the standard deviation of power across the 100 replication data sets. These results are given in the Additional file 1.

#### Power (definition 1) to detect interacting SNPs for a fixed significance threshold

Although the statistical significance level is unreliable for measuring performance of the methods (as illustrated in Table 1), we want to give readers an empirical sense of how the methods perform when using the statistical significance level to select candidate SNPs in the step 2 experiment. These results, given in the Additional file 1, show that using the same significance threshold, the methods detect very different numbers of both true positive and false positive SNPs. Moreover, considering the balance of true positives and false positives achieved by each of the methods, none of them performs strongly.

#### Power to detect entire interactions (definition 2)

**Figure 7:**
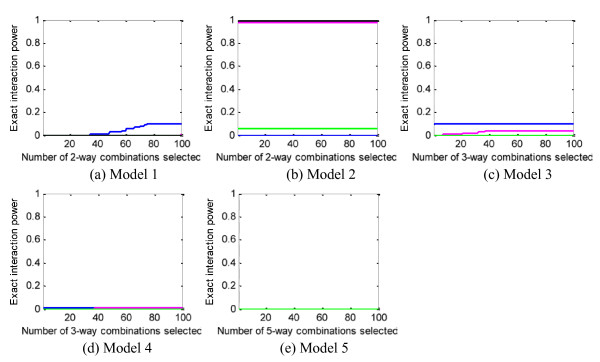
**Power evaluation (*definition 2*) of the methods on 100 replication data sets with parameter setting: θ = 1.4, *β *= 1, *l *= null**. In (a), FIM, IG, MDR and LRIT have power constantly equal to 0; in (b) FIM and IG and LRIT have power constantly equal to 1; in (d) SH, FIM and MDR have power constantly equal to 0. Blue curve - SH, magenta curve - FIM, green curve - MDR, black curve - IG, grey curve - LRIT, yellow curve - LR.

Based on power *definition 2*, we did experiments to evaluate all the methods on the 1000-SNP data sets of Step 2. Considering the high computational complexity and the applicability of the methods, we compare the power of IG, LRIT, FIM, SH and MDR on 2-way interactions, the power of FIM, SH, and MDR on 3-way interactions, and the power of MDR on 5-way interactions. Figure 7 shows the results. Due to the limited number of *total *interactions output by BEAM and MECPM, we do not evaluate BEAM here, and list the power of MECPM only at its stopping point: model 1 - 0, model 2 - 0.96, model 3 - 0.94, model 4 - 0, model 5 - 0.46.

We can observe that all the methods have poor performance for models 1 and 4. For models 3 and 5, all the methods fare poorly except for MECPM. For model 2, IG, LRIT and FIM have very good performance (power = 1); MECPM also performs well (power = 0.96); while the other methods still perform poorly.

#### Power to detect at least 1 SNPin an interaction - partial interaction detection (definition 3)

**Figure 8:**
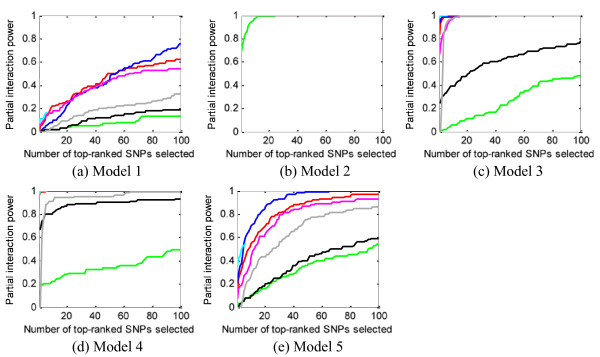
**Power evaluation (*definition 3*) of the eight methods on 100 replication data sets with parameter setting: θ = 1.4, *β *= 1, *l *= null**. Blue curve - SH, magenta curve - FIM, green curve - MDR, black curve - IG, grey curve - LRIT, yellow curve - LR.

Based on power *definition 3*, we evaluated SH, BEAM, IG, FIM, MDR, LRIT and MECPM. The major results are shown in Figure 8. Due to the limited number of *total *interactions output by MECPM at its stopping criteria, we give a text description, instead of drawing a curve, to show the power at its stopping point: model 1 - 0.17, model 2 - 1, model 3 - 0.98, model 4 - 0.97, model 5 - 0.46. From Figure 8, BEAM, SH, FIM, LRIT and MECPM obtain good results for models 2, 3, 4, 5. We believe that these good results are partly due to the relatively strong main effects of SNPs involved in these interaction models. Note that there is a substantial increase in power compared to Figure 7. Also, by comparing with the results for power *definition 1 *(Figure 4), we can see that there is largely increased power for most models, indicating most interaction models can be partly detected by the methods.

#### Power to detect individual SNP main effects (definition 4)

**Figure 9:**
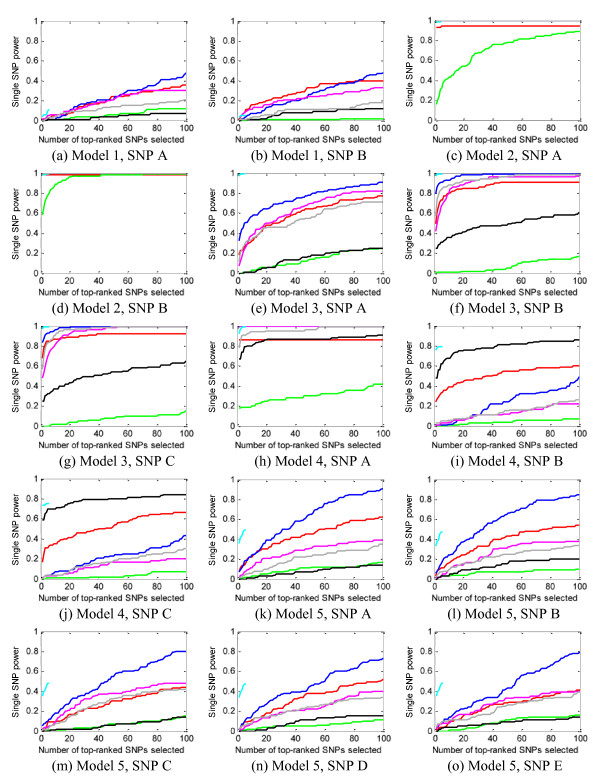
**The power to detect individual SNPs, for parameter *θ *= 1.4, *β *= 1, *l *= null**. Blue curve - SH, magenta curve - FIM, green curve - MDR, black curve - IG, cyan curve -MECPM, grey curve - LRIT, yellow curve - LR.

Based on Figure 9, we can confirm our previous statement that main effects play an important role in determining whether or not a SNP can be detected. For example, the two SNPs in model 2 (odds ratio: 1.89 in the basic model) and SNP A (odds ratio: 2.45 in the basic model) in model 4 have strong main effects, and all the methods detect them well.

Also, we observe similar power for SNPs participating in interactions with symmetric penetrance tables and the same MAFs. For example, all the SNPs in model 1 and model 5 have similar power; likewise for SNPs *B *and *C *in models 3 and 4. This observation is reasonable since these SNPs not only have the same main effects, but also have the same interaction effects.

For SNPs participating in interactions with a symmetric penetrance table but different MAFs, an interesting (and perhaps unexpected) finding is that for model 2, the power to detect SNP *A *(MAF = 0.2), is *greater than *the power to detect SNP *B*, which has a larger MAF (MAF = 0.3). We give theoretical justification for this result in section 1 of the Additional file 1.

#### Performance for step 3, the main-effect-only case

**Figure 10:**
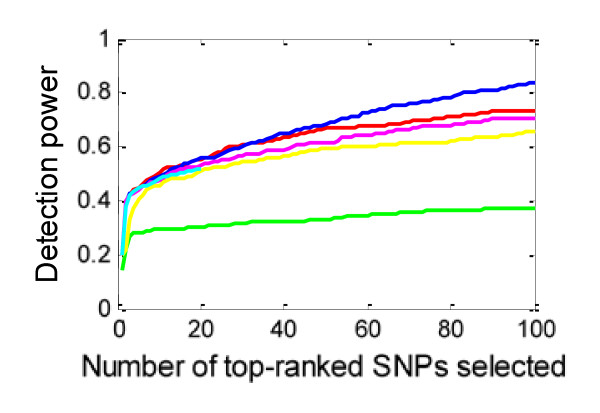
**Power evaluation of 6 methods (using power *definition 1*) on main-effects-only data (step 3)**. Blue curve - SH, magenta curve - FIM, green curve - MDR, cyan curve - MECPM, yellow curve - LR.

We used power *definition 4 *to evaluate performance of the methods on the main-effect-only data sets in Step 3. We did not include the IG and LRIT method in this Step because IG and LRIT only detect multilocus interactions, not single (main effect) SNPs; thus, for Step 3, involving only main effects, detected interactions, even ones involving the main effect SNPs, are necessarily false positive interactions. Figure 10 shows the power curves, from which we observe that, except for MDR, most methods (FIM, BEAM, SH, MECPM, LR) achieve similar, good power at the beginning, with SH becoming a bit better as *K *increases.

We also evaluated whether the methods detect false positive interactions when there are *only *main effects. Here we evaluated the 3 methods that give P-value assessments, looking at the number of false positive interactions detected under the P-value of 0.1 after Bonferroni correction. Table 2 lists the results, from which we can see that BEAM and SH are quite good at inhibiting false positive interactions caused by marginal effects, but FIM produces many false positive interactions.

**Table 2:** The average number of false positive interactions (step 3) for BEAM, SH and FIM under the significance threshold of 0

number of false positives	BEAM	SH	FIM
2nd order	0	0	2.21

3rd order	0	0	64.19

#### Computational complexity and memory occupancy

**Figure 11:**
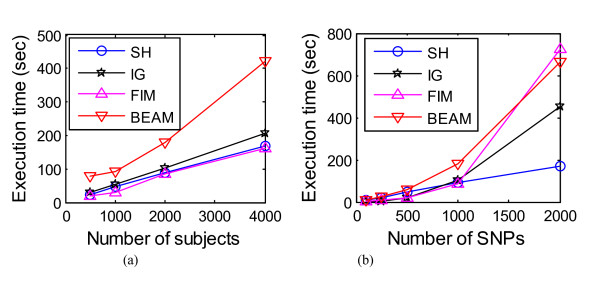
**Execution time (sec) of 4 methods for: (a) number of SNPs = 1,000; (b) number of subjects = 2,000**. Due to limited space in (b), we list hereby the execution time of the methods on 2000-subject 10,000-SNP data: SH - 962 seconds, IG - 18291 seconds, BEAM - 36423 seconds, FIM - 91251 seconds.

Computational complexity for the eight methods was evaluated for the same platform: OS: Windows, CPU: 3G, RAM: 2G. SH, IG, FIM, LR, LRIT, MECPM and BEAM do not require much memory, but the exhaustive search used by MDR requires an impractical amount of memory for a large number of SNPs. Thus, as noted earlier, we applied the heuristic search option in the MDR software, with a 1 hour time limit to avoid memory overflow. Figure 11(a) shows that, as expected, most methods' execution times increase linearly with sample size. The exception is BEAM execution, which grows more quickly. Figure 11(b) shows execution times for different numbers of SNPs. SH obtains the highest efficiency (~ linearly increasing execution time); IG and BEAM are more time consuming (~ quadratically increasing); and FIM is most time-consuming (~ cubic in the number of SNPs). Besides Figure 11(b), we also list execution time of LR, LRIT and MECPM (at MECPM's stopping point): the execution time of LR on 1000-SNP data and 10000-SNP data is 1 second and 10 seconds, respectively; the execution time of LRIT on 1000-SNP data and 10000-SNP data sets is 24 seconds and 576 seconds, respectively; the execution time for MECPM on the 1000-SNP data and 10,000 SNP data was 7033 seconds and 25944 seconds, respectively. Compared with Figure 11(b), we can see that MECPM's computation complexity is, relatively, quite high for 1000 SNPs, but is in fact lower than that of several of the other methods for 10,000 SNPs.

## Discussion

### General Summary of the Study and Its Results

We report a comparison of eight representative methods, multifactor dimensionality reduction (MDR), full interaction model (FIM), information gain (IG), Bayesian epistasis association mapping (BEAM), SNP harvester (SH), maximum entropy conditional probability modeling (MECPM), logistic regression with an interaction term (LRIM), and logistic regression (LR). The first seven were specifically designed to detect interactions among SNPs, and the last is a popular main-effect testing method serving as a baseline for performance evaluation. The selected methods were compared on a large number of simulated data sets, each, consistent with complex disease models, embedded with *multiple *sets of interacting SNPs, under different interaction models. The assessment criteria included several relevant detection power measures, family-wise type I error rate, and computational complexity. The principal experimental results are as follows: i) while some SNPs in interactions with strong effects are successfully detected, most of the methods miss many interacting SNPs at an acceptable rate of false positives; in this study, the best-performing method was MECPM; ii) the statistical significance assessment criteria, used by some of these methods to control the type I error rate, are quite conservative, which further limits their power and makes it difficult to fairly compare them; iii) the power varies for different models as a function of penetrance, minor allele frequency, linkage disequilibrium and marginal effects; iv) analytical relationships between power and these factors are derived, which support and help explain the experimental results; v) for these methods the magnitude of the main effects plays an important role in whether an interacting SNP is detected; vi) most methods can detect some ground-truth SNPs, but fare modestly at detecting the whole set of interacting SNPs.

### Based on the simulation data sets used in this study, which include multiple interaction models present in each data set in Step 2, most of the methods miss some interacting SNPs, leading to only moderate power at low false positive SNP counts (Figures 4, 5)

Compared to the promising powers achieved for the simulation studies reported in the methods' respective papers, the degraded performance seen in this comparative study for most methods is attributed to the more difficult yet likely more realistic simulation data that we used. The methods (excepting LR and MECPM) were previously reported as powerful on simulation data sets including only a single, strong ground-truth interaction, but our study included 5 interactions present in each data set to simulate multiple genetic causes for complex diseases. The disease risk is thus effectively divided among the 5 interaction models, giving each a weaker (less easily detected) effect.

### Main effects play an important role in whether a ground-truth SNP is detected at low false positive SNP counts

Another notable finding is that the main effects of the interacting SNPs affect their likelihood of being detected at low false positive SNP counts by most methods. For interaction models with very weak marginal effects (models 1 and 5), all the methods have low power (see Figure 4(b) and 4(f)). Although some methods (e.g. SH) emphasize the detection of interactions with weak marginal effects, their results on these models are very modest. Heuristic search strategies used by the methods count on at least one SNP in the interaction having a relatively strong effect; this explains why model 1, without strong main effects, is difficult to detect. Moreover, the huge search space for 5-way interactions makes it easy for heuristic search strategies to miss model 5.

### For the same interaction model, different levels of power are achieved by the eight methods

For each interaction model, the power varies across methods because of the quite different detection principles applied by the methods. For example, IG and LRIT, which are based on pairwise SNP statistics, can detect 2-way interaction effects well (see models 2 and 4, where model 4 can be considered as two overlapped 2-way interactions), but IG and LRIT gets poorer results for higher-order models. For the difficult 5-way interaction, only MECPM gave promising results.

### Power on the whole ground-truth SNP set - MECPM performs the best, while MDR performs the worst

From Figure 4(a), MECPM achieves the best performance; BEAM, SH, FIM, LR, LRIT and IG have similar and moderate performance; MDR performs the worst, among the eight methods we tested. From Figure 6, SH outperforms BEAM and FIM for weaker effects (i.e., for discounted penetrance values and MAF). Here we briefly discuss how these performance differences are a product of the different methodologies employed.

Power may be degraded by an insufficiently sensitive ranking criterion, by the heuristic search strategy used, or by a suboptimal output design of a method. The high computational complexity of MDR necessitates using its heuristic search option to keep the running time/memory usage in a reasonable range. This heuristic search forces a significantly reduced search space, and hence the performance of MDR is expected to be degraded.

The ranking criterion of IG detects pure interaction effects (see equation (4) and the definition of mutual information). However, what really affects disease risk is a combination of both pure interaction effects and main effects. Additionally, IG is only explicitly designed to detect 2-way interactions, and thus may have difficulty detecting higher order ones.

Comparatively, MECPM, BEAM, FIM and SH have less critical limitations, with these mainly in the sensitivity of their ranking criteria and their use of heuristic search -- e.g., the difficulty for heuristic search to pick up interactions with weak marginal effects and high-order interactions due to the large search space (Consider a contingency table with 3^5 ^= 243 cells for a 5-way interaction.).

### The performance of the methods is sensitive to changes in penetrance value, MAF, and LD

From Figure 6, the seven methods all have clearly decreased power when we reduce penetrance values and the MAF, or replace ground-truth SNPs by surrogates in LD with them. Among the methods, SH is the most robust while IG is the most sensitive to these factors. Besides our empirical results, a theoretical analysis of how power changes with penetrance or MAF is given in the Additional file 1. The analytical results, which are consistent with (and thus support and explain) our experimental results are as follows: 1) increasing the penetrance of an interaction model results in both a stronger (more easily detected) joint interaction effect and in stronger marginal effects of the participating SNPs; 2) increasing the frequency of a disease-related genotype results in a stronger joint effect, under certain conditions; 3) the impact of genotype frequency on main effects is more complicated -- when the marginal frequency, *a*, of a disease-related genotype is small, the strengths of the marginal effects increase when *a *increases, and when *a *is large, the strengths of the marginal effects *decrease *as *a *increases.

### Most methods can partially but not exactly detect the interactions

The results for power *definition 2 *(see Figure 7) are quite different from those for power *definition 1 *(see Figure 4), indicating that most methods detect the interacting ground truth SNPs as singlets or subsets of the ground truth interactions. There are multiple reasons for this, in some cases method-specific. For example, the large degrees of freedom of FIM render a high false positive rate, making ground-truth interactions easily buried amongst many false positives; due to the use of heuristic search strategies, the methods may not even *evaluate *the ground-truth interactions as candidates; also, for some methods, e.g. FIM, successful detection of an interaction relies on *first *detecting main effects for (at least some) SNPs involved in the interaction, thus this type of heuristic search strategy will miss ground-truth interactions that possess only weak main effects; moreover, SH *excludes *SNPs with strong main effects from higher-order search, so SH in particular will miss interactions that possess *strong *main effects (see Figure 7(b)).

### The P-value assessments of BEAM, SH and FIM are variable across method and all are overly conservative

From the subsection "*Power for a fixed significance threshold*" and results given in the Additional file 1, we observe that for the same significance threshold, BEAM, SH and FIM have quite different power and false positive SNP counts. Also, in the subsection "*Accuracy of P-value assessment in step 1*", we showed that their P-value assessments are conservative for 2nd and 3rd order interactions. From further experiments, we conclude that this phenomenon originates from three factors: the heuristic search strategies, dependencies between SNP combinations, and the summary statistics used by the methods.

For BEAM, SH, and FIM, the heuristic search strategies evaluate fewer SNP combination candidates than the number actually penalized in the Bonferroni correction. Moreover, SH and BEAM exclude SNPs with strong marginal effects from high-order interactions, which further decreases the number of searched SNP combinations. So the Bonferroni-corrected P-value is smaller than it should be. Also, some SNP combinations have dependencies with others, either because they share a common SNP subset and/or because SNPs in different subsets are in LD. Such dependencies make the Bonferroni correction inherently conservative.

Besides heuristic search and dependencies, the conservativeness also derives from the summary statistics themselves. The authors of BEAM evaluated the B statistic's conservativeness with exhaustive search. In the Additional file 1, we likewise evaluate conservativeness of the *χ*^2 ^statistics applied by SH and FIM. We considered the case where there is neither multiple testing nor heuristic search. The *χ*^2 ^statistics turn out to be conservative, becoming more so as the significance threshold is decreased (see Tables 1, 2 in the Additional file 1). Theoretically, such conservativeness may come from the discreteness of the SNP data. Since the *χ*^2 ^statistics in SH and FIM are calculated from the discrete-valued SNP data, the *χ*^2 ^statistics are also discrete. At the tail part of the *χ*^2 ^distribution, two consecutive discrete *χ*^2 ^values may correspond to very different significance levels. For example, let the P-values of consecutive *χ*^2 ^values be *p*_1_, *p*_2 _(*p*_1 _>>*p*_2_); when the significance threshold is *p*_0 _and *p*_1 _>*p*_0 _>*p*_2_, the type I error rate actually corresponds to *p*_2_, which is much less than *p*_0_, making the results quite conservative.

### Limitations of the Current Study and Future Work

There are a number of possible extensions of this simulation study that we intend to consider in our future work. First, our current simulation software only handles categorical traits and categorical (ternary-valued, SNP) covariates. Environmental covariates and admixture-adjusting variables could be either quantitative or ordinal-valued. Likewise, traits (phenotype) could be quantitative or ordinal. There are natural ways of extending our current simulation approach to allow for these more general covariate and trait types, which we will consider in future work. Second, we have not investigated missing SNP-values and their effect on detection power. Third, while we have chosen five plausible penetrance function models, another possibility would be to use "data-driven" penetrance functions, *i.e*. penetrance functions estimated based on real GWAS data sets with known ground-truth and known (*i.e*., previously detected) interactions.

## Conclusions

The methods explored in this study are useful tools in the exploration of potential interacting loci. Each of the methods studied here has its strengths and weaknesses. Our comparative examination of these methods suggests that continued research into methods that test for interacting loci is necessary to expand the tools available to researchers and to achieve improved power for detecting complex interactions, along with accurate assessment of statistical significance.

## Methods

### Methods Tested in the Comparison Study

The eight [32-35,39-41] methods originate from different underlying techniques and principles, and thus can be categorized in different ways, as shown in Table 3. FIM, BEAM, SH, LRIT, and LR asymptotically approximate the null distribution to assess statistical significance; MECPM models SNP interactions under a maximum-entropy principle, and uses the Bayesian information criterion (BIC) as the model selection strategy; MDR and IG only provide a ranking of candidate interactions. These methods employ three main search strategies: exhaustive search (IG, LRIT and LR), stochastic search (BEAM and MDR), and deterministic heuristic search (SH, FIM and MECPM). Each method uses a different detection principle: SH applies *χ*^2 ^or *B *statistics [32,39]; BEAM uses Bayesian inference or B statistics; FIM, LRIT and LR are based on the logistic regression model; IG ranks SNPs by mutual information; MDR selects SNPs via prediction error; MECPM uses BIC to rank interactions and to assess statistical significance.

**Table 3:** Properties of methods tested in this paper.

Name	Detection Principle	Heuristic search	Asymptotic null distribution	Free-accessible software
MDR	Prediction accuracy	Stochastic	No	http://www.multifactordimensionalityreduction.org/

FIM	Logistic regression	Deterministic	Yes	N/A

IG	Mutual Info.	N/A	No	N/A

BEAM	Bayesian model	Stochastic	Yes	http://www.fas.harvard.edu/~junliu/BEAM/

MECPM	BIC	Deterministic	No	http://www.cbil.ece.vt.edu/software/MECPM.zip

SH	χ^2^or *B *statistic	Deterministic	Yes	http://bioinformatics.ust.hk/SNPHarvester.html

LRIT	Logistic regression	N/A	Yes	N/A

LR	Logistic regression	N/A	Yes	N/A

For the methods without free-accessible software by the authors, we provide our self-written software, as well as C++ code, at http://code.google.com/p/simulation-tool-bmc-ms9169818735220977/downloads/list

A brief summary of these eight methods follows.

#### (1) Multifactor dimensionality reduction (MDR) [33]

For a set of SNPs, MDR labels a genotype as "high-risk" if the ratio between the number of cases and the number of controls exceeds some threshold (e.g., 1.0). A binary variable is thus formed, pooling high-risk genotypes into one group and low-risk ones into another. If the subject has a high-risk genotype it is predicted as a case; otherwise as a control. The prediction error of each model is estimated by 10-fold cross validation and serves as the measure of association between the set of SNPs and the disease.

#### (2) Full Interaction Model (FIM) [41]

In FIM, 3*^d^*-1 binary variables *x_j_, j *= 1,2,..., 3*^d^*-1 are introduced for a subset of *d *SNPs and a logistic regression model with 3*^d ^*parameters is estimated from the data. *x_j_*(*i*) corresponds to the *jth *genotype combination (or interaction term) of the SNP subset on the *ith *subject. *x_j_*(*i*) = 1 if the *jth *genotype combination is present for the *ith *subject, and 0 otherwise. For the row vector , let *π*(**x**(*i*)) be the disease risk. The logistic regression is parameterized as(3)

where  are estimated via maximum likelihood estimation. A likelihood-ratio test is applied to calculate the significance of this SNP subset via an asymptotic *χ*^2 ^distribution.

#### (3) Information Gain (IG) [34]

Let *C *denote the disease status random variable. The information gain of {*A, B*} is defined as(4)

where the mutual information *I*(*A*;*B*) is a non-negative measure of the reduction in uncertainty about the value of (SNP locus) random variable *A*, given knowledge of random variable *B *[61] Equivalently, it is a measure of the statistical dependence between *A *and *B*. The conditional mutual information *I*(*A*;*B |C*) likewise gives a measure of the statistical dependence between *A *and *B *given that the phenotype random variable, *C*, is known. The magnitude of IG thus indicates the increased statistical dependence between *A *and *B *given knowledge of *C, i.e*. the strength of an interaction between loci *A *and *B*.

#### (4) Bayesian Epistasis Association Mapping (BEAM) [32]

Suppose *N *samples (*N_d _*cases and *N_u _*controls) are genotyped at *L *SNPs. BEAM partitions the *L *SNPs into 3 groups: markers with no association with disease, markers with only main effects, and markers with interaction effects. Let the genotypes on cases be *D *= (*d*_1_,...,*d_L_*) with  representing genotype vector of the *j*th SNP at all the cases. According to the abovementioned partitioning, *D *can be divided into three subsets, *D*_0_, *D*_1_, and *D*_2_, where *D*_0 _is the subset consisting of SNPs (SNP genotype vectors) with no association, *D*_1 _is the subset consisting of SNPs with only main effects, and *D*_2 _is the subset consisting of SNPs with interaction effects. Likewise, let the genotypes on controls be *U *= (*u*_1_,...,*u_L_) *with  representing genotypes of the *j*th SNP at the controls. Let **I **= [*I*_1_,*I*_2_,..., *I*_L_] be the membership of SNPs within each group, e.g. *I_j _*= 0 means that the *jth *SNP has a main effect, *I_j _*= 1 means that the *jth *SNP has only main effects, *I_j _*= 2 means that the *jth *SNP has interaction effects. Let *P*() be the probability symbol. Following some assumptions [32], the posterior distribution of **I **given *D *and *U *is inferred by:(5)

Based on equation (5), BEAM draws **I **using the Metropolis-Hastings algorithm. The output is the posterior probability of main-effect markers and interactions associated with the disease. A "*B*" statistic is also applied to measure statistical significance of SNPs and interactions.

#### (5) SNP Harvester (SH) [39]

This method aims to detect interactions with weak marginal effects. It includes the following steps:

5a. Remove SNPs with significant main effects;

5b. For a fixed *M*, run the "PathSeeker" heuristic search to identify *M*-way SNP interactions. First, randomly select *M *SNPs to form a *M*-way set A = {*x*_1_, *x*_2_,..., *x_M_*}. Second, swap one of the remaining SNPs with each member of **A**, to see whether a statistical score *s*(**A**) (e.g. χ^2^statistic, *B *statistic) increases. Then iteratively repeat this second step until convergence; record *s*(**A**) if statistically significant. Then go back to the first step, with the optimal **A **removed as a candidate for the next run.

5c. Use L2-norm penalized logistic regression [37] as a post processing step to further select interactions from those identified in 5b.

Although SH *removes *SNPs with strong main effects, for purpose of fair comparison, we still give it credit for identifying these main-effect SNPs in calculating its power.

#### (6) Maximum entropy conditional probability modeling (MECPM) [40]

MECPM builds the phenotype posterior under a maximum entropy principle, encodes constraints into the model that correspond 1-to-1 to interactions, flexibly allows dominant or recessive coding for each locus in a candidate interaction, searches interactions via a greedy interaction growing search strategy that evaluates candidates up to fifth order, and uses the Bayesian information criterion (BIC) as the model selection strategy.

#### (7) Logistic regression (LR) [35]

LR is a generalized linear model used for binomial regression. Let *x*(*i*) correspond to the genotype of a SNP for the *ith *subject. *x*(*i*) = 0 denotes homozygous major alleles; *x*(*i*) = 1 denotes heterozygous genotypes; and *x*(*i*) = 2 denotes homozygous minor alleles. Let *π *(*x*(*i*)) be the disease risk. The logistic regression is parameterized as:(6)

, where *β*_0 _and *β*_1 _are the regression coefficients, learned via maximum likelihood. By a likelihood ratio test, logistic regression evaluates statistical significance for each SNP.

#### (8) Logistic regression with interaction term (LRIT) [35]

LRIT aims at detecting interaction effects based on the logistic regression model. Let *x_m_*(*i*) and *x_n_*(*i*) correspond to genotypes of the *mth *SNP and *nth *SNPs for the *ith *subject, respectively. *x_m_*(*i*) = 0 or *x_n_*(*i*) = 0 denotes homozygous major alleles; *x_m_*(*i*) = 1 or *x_n_*(*i*) = 1 denotes heterozygous genotypes; and *x_m_*(*i*) = 2 or *x_n_*(*i*) = 2 denotes homozygous minor alleles. Let *π *(*x_m_*(*i*), *x_n_*(*i*)) be the disease risk. The logistic regression is parameterized as:(7)

, where *β*_0_, *β*_1_, *β*_2_, *β*_3 _are the regression coefficients, learned via maximum likelihood. By a likelihood ratio test, logistic regression evaluates the statistical significance for this pair of SNPs (the statistical significance reflects the joint effects of the two individual terms and the multiplicative term).

## Authors' contributions

LC and YW designed the experiment protocols and evaluation measures, conducted the experiments, participated in implementation of the methods and design of simulation tools, and drafted the manuscript. GY implemented the conventional (also some advanced) interaction-detection methods, and participated in design of the experiments. CL designed the simulation tools. CL, RG and XY carried out the development of simulation software. DM helped to draft and extensively edited the manuscript. DM and JR implemented MECPM. YW and DH conceived of the study, participated in its design and coordination, and helped draft the paper. All authors read and approved the final manuscript.

## Supplementary Material

Additional file 1 **Supplementary information: comparative analysis of methods for detecting interactive SNPs**. This supplementary information consists of 6 sections: S1. Section S1 presents our theoretical analysis of the relationship between association strength, joint effect, main effect, penetrance function, and MAF. This section also provides some theoretical explanations about our experimental results. S2. Section S2 presents comprehensive power evaluation results of the methods for different interaction models and parameter settings, related to power *definition 1*. The reproducibility of the methods is also shown by the standard deviation of power. As an extension of the main text, we also summarize our findings and analytical explanations for these results. S3. Section S3 provides ROC curves of the methods based on the whole ground-truth SNP set. These ROC curves illustrate the sensitivity and specificity for the methods. The reproducibility of the methods is also shown by the standard deviation of sensitivity. S4. Section S4 describes in detail how the effect size (odds ratio) is calculated for each interaction model. S5. Section S5 analyzes the conservativeness of χ^2 ^statistics applied by SH and FIM. This analysis partly explains why SH and FIM are conservative. S6. Section S6 gives the empirical relationship between power and the false positive SNP count under a given significance threshold. Click here for file
