# Supplementary material for: Comparative analysis of methods for detecting interacting loci
Source: BMC Genomics. 2011 Jul 5;12:344. doi: 10.1186/1471-2164-12-344 (PMC3161015; doi:10.1186/1471-2164-12-344)
Supplement: Additional file 1 — Supplementary information: comparative analysis of methods for detecting interactive SNPs. This supplementary information consists of 6 sections: S1. Section S1 presents our theoretical analysis of the relationship between association strength, joint effect, main effect, penetrance function, and MAF. This section also provides some theoretical explanations about our experimental results. S2. Section S2 presents comprehensive power evaluation results of the methods for different interaction models and parameter settings, related to power definition 1. The reproducibility of the methods is also shown by the standard deviation of power. As an extension of the main text, we also summarize our findings and analytical explanations for these results. S3. Section S3 provides ROC curves of the methods based on the whole ground-truth SNP set. These ROC curves illustrate the sensitivity and specificity for the methods. The reproducibility of the methods is also shown by the standard deviation of sensitivity. S4. Section S4 describes in detail how the effect size (odds ratio) is calculated for each interaction model. S5. Section S5 analyzes the conservativeness of χ2 statistics applied by SH and FIM. This analysis partly explains why SH and FIM are conservative. S6. Section S6 gives the empirical relationship between power and the false positive SNP count under a given significance threshold. [file 1471-2164-12-344-S1.DOC]

Supplementary Information

Comparative Analysis of Methods for Detecting Interactive SNPs

Li Chen^1^, Guoqiang Yu^1^, Carl D Langefeld^2^, David J Miller^3^, Richard T. Guy^2^, Jayaram Raghuram^3^, Xiguo Yuan^1^, David M Herrington^4^, and Yue Wang^1,*^

1 Bradley Department of Electrical and Computer Engineering, Virginia Polytechnic Institute and State University, Arlington, VA, USA

2 Department of Biostatistical Sciences, Wake Forest School of Medicine, Winston-Salem, NC, USA

3 Department of Electrical Engineering, The Pennsylvania State University, University Park, PA, USA

4 Department of Internal Medicine, Wake Forest School of Medicine, Winston-Salem, NC, USA

**Introduction**

This supplementary information consists of 6 sections:

S1. Section S1 presents our theoretical analysis of the relationship between association strength, joint effect, main effect, penetrance function, and MAF. This section also provides some theoretical explanations about our experimental results.

S2. Section S2 presents comprehensive power evaluation results of the 7 methods for different interaction models and parameter settings, related to the power definition 1. The reproducibility of the methods is also shown by the standard deviation of power. As an extension of the main text, we also summarize our findings and analytical explanations for these results.

S3. Section S3 provides ROC curves of the methods based on the whole ground-truth SNP set. These ROC curves illustrate the sensitivity and specificity for each method. The reproducibility of the methods is also shown by the standard deviation of sensitivity.

S4. Section S4 describes in detail how the effect size (odds ratio) is calculated for each interaction model.

S5. Section S5 analyzes the conservativeness of $\chi^{2}$statistics applied by SH and FIM. This analysis partly explains why SH and FIM are conservative (i.e., empirical false positive rates are overestimated).

S6. Section S6 gives the empirical relationship between power and the false positive SNP count under a given significance threshold.

**S1. Theoretical analysis on the relationship between joint effect, main effect, penetrance function and MAF**

In section S1.1, we theoretically analyze how the penetrance function and MAF affect the statistical significance of an interaction model. In section 1.2, we show that our theoretical conclusions support our experimental results under various power definitions.

**S1.1 Theoretical analysis on the relationship between statistical significance, penetrance function, and MAF of an interaction model**

Here we present our mathematical inference on how the penetrance function and MAF affect the statistical significance of an interaction model.

*S1.1.A. An approximate yet general interaction model*

For a combination of interacting SNPs, let $g$ denote its genotype for a patient, and $d$ be the disease status (if the patient has the disease, $d=1$; otherwise$d=0$). For ease of analysis, we dichotomize the genotypes of this SNP combination into a set of disease-related genotypes $\mathbf{G}$ and a set of disease-unrelated genotypes: when $g\in\mathbf{G}$, the disease risk brought by the interaction model is $m$; when $g\in\mathbf{G}$, the disease risk brought by the interaction model is $0$ .

We consider a complex yet realistic situation: the disease has multiple SNP-SNP interaction causes (factors), as well as other unknown genetic/environmental factors. For simplicity, we treat the current interaction model as one cause for the disease and effectively treat all the other factors lumped together as another independent cause, *i.e.,* the second cause will provide a baseline disease risk $q$, regardless of whether $g\in\mathbf{G}$ or not. Since these two causes independently affect the disease status, a non-disease status is obtained only when *neither* of the two causes leads to the disease. Thus, we have

$\begin{matrix} \text{Prob}(d=0|g\in\mathbf{G})=(1-m)\cdot(1-q) \\ \text{Prob}(d=0|g\notin\mathbf{G})=(1-0)\cdot(1-q)=1-q \end{matrix}$,

and accordingly

$\begin{matrix} \left\{\begin{matrix} \text{Prob}(d=1|g\in\mathbf{G})=m+q-mq \\ \text{Prob}(d=1|g\notin\mathbf{G})=q \end{matrix}\right. \\ \Rightarrow\left\{\begin{matrix} \text{Prob}(d=1,g\in\mathbf{G})=\text{Prob}(d=1|g\in\mathbf{G})\cdot\text{Prob}(g\in\mathbf{G})=B(m+q-mq) \\ \text{Prob}(d=1,g\notin\mathbf{G})=\text{Prob}(d=1|g\notin\mathbf{G})\cdot\mathrm{P}\text{rob}(g\notin\mathbf{G})=(1-B)q. \end{matrix}\right. \end{matrix}$ (1)

Let $k$ be the observed number of cases with $g\in\mathbf{G}$ and $N$ be the total number of subjects. From equation (1), the expectation of $k$ is given by $N(m+1-mq)$. Letting $\eta$ be the ratio between the number of cases and that of controls, we can construct the contingency table in Table 1.

**Table 1.** Contingency table of the interaction model defined in (1)

|  | $g\in\mathbf{G}$ | $g\notin\mathbf{G}$ | Column sum |
| --- | --- | --- | --- |
| Disease | $k$ | $N\eta-k$ | $N\eta$ |
| Normal | $NB-k$ | $N(1-B)-\eta N-k$ | $N(1-\eta)$ |
| Row sum |  | $N(1-B)$ | $N$ |

From Fisher’s Exact Test, we can estimate the statistical significance of this observation based on the hypergeometric distribution$K\sim H(N,NB,N\eta)$. The P value is given by

$\text{Pval=Prob}(K>k|B,\eta,N)$.

Note that this hypergeometric distribution is the null distribution, and the observation $k$ is generated by the alternative hypothesis denoted by (1). We then consider the ideal case where the observations are equal to their expectation based on the alternative hypothesis, *i.e.,* from (1), $k=NB(m+1-mq)$ and $N\eta-k=N(1-B)q$, and the P value becomes $\text{Pval=Prob}(K>N(m+1-mq)|B,\eta,N)$. Since $k=NB(m+1-mq)$ and $N\eta-k=N(1-B)q$, we can obtain

$\eta=Bp+(1-B)q$, (2)

where $p=m+(1-m)q$.

From the definition of hypergeometric distribution, the expectation of $K$ (based on the null distribution) is

$$\text{E}[K]=NB\eta=NB[Bp+(1-B)q]$$

, and the variance of $K$ is

$\begin{matrix} \text{Var}\left(K\right)=N^{2}B(1-B)[Bp+(1-B)q][B(1-p)+(1-B)(1-q)]/(N-1) \\ \approx NB(1-B)[Bp+(1-B)q][B(1-p)+(1-B)(1-q)] \end{matrix}$.

**Direct estimation of the P value requires us to calculate the probability mass function at every possible value of $K$, which is computationally hazardous and too complex for our further analysis.** **So we approximate$K$’s hypergeometric distribution by a Gaussian distribution:**

$$K\sim G(NB[Bp+(1-B)q], NB(1-B)[Bp+(1-B)q][B(1-p)+(1-B)(1-q)])$$

This approximation is good when 3 conditions are met: i) $N$ is large enough; ii)$N$ is large compared to $NB$; iii) $Bp+(1-B)q$is not close to 0 and 1. For GWAS, $N$ is usually large enough, so i) is met; and in most cases ii) and iii) can also be hold. Moreover, our subsequent analysis focuses on the increasing/decreasing relationship between power and different parameters, instead of the accurate quantitative measurement, so we use this approximation.

After standardization of $K$, we have

$X=\frac{K-NB[Bp+(1-B)q]}{\sqrt{NB(1-B)[Bp+(1-B)q][B(1-p)+(1-B)(1-q)]}}\sim G(0,1)$.

So the approximated P value is given by

$$\text{Prob}(K>NBp|B,p,q,N)=\text{Prob}(X>x_{0})=\text{erfc(}x_{0}\text{)}$$

, where erfc is the complementary Gaussian error function and

$\begin{matrix} x_{0}=\frac{NBp-NB[Bp+(1-B)q]}{\sqrt{NB(1-B)[Bp+(1-B)q][B(1-p)+(1-B)(1-q)]}} ∵p=m+(1-m)q \\ =\frac{\sqrt{N}\cdot\sqrt{B(1-B)}\cdot\sqrt{1-q}}{\sqrt{q}\cdot\sqrt{1/m+B/q-B}\cdot\sqrt{1/m-B}} \end{matrix}$. (3)

*S1.1.B. The relationship among statistical significance, penetrance, and the frequency of disease-related genotypes of an interaction model.*

First we consider the general relationship between the P value of the interaction model, the penetrance value$m$, and the frequency of the disease-related genotypes $B$.

With fixed$B$, from (3), we can see that when $m$ become larger, $\sqrt{1/m+B/q-B}$ and $\sqrt{1/m-B}$ both will become smaller; thus $x_{0}$ will become larger. Since erfc is a decreasing function, the P value $\text{erfc(}x_{0}\text{)}$ will become smaller. **Thus, the P value of the interaction model is decreasing for increasing penetrance value $m$.**

With fixed $m$, equation (3) can be rewritten as;

$\begin{matrix} x_{0}=\frac{NBp-NB[Bp+(1-B)q]}{\sqrt{NB(1-B)[Bp+(1-B)q][B(1-p)+(1-B)(1-q)]}} ∵p=m+(1-m)q \\ =\sqrt{N}\cdot\frac{\sqrt{B(1-B)}}{\sqrt{B+q/(m-mq)}\cdot\sqrt{1/m-B}} \end{matrix}$ (4)

**The factor $\frac{\sqrt{B(1-B)}}{\sqrt{B+q/(m-mq)}\cdot\sqrt{1/m-B}}$ is quite complicated and not necessarily monotonic, so the relationship between P value and $B$ is not so simple.** Here, we give some hints on how $B$ affects the P value in **extreme cases**. Since $B$ is the frequency of genotypes, $0\leq B\leq1$. When $B=0$ or $B=1$, from (4), we get$x_{0}=0$, which means the interaction effect is not significant at all. **Therefore, when the frequency of the disease-related genotypes is either too large (**$B=1$**) or too small (**$B=0$**), the interaction effect will be very small. Intuitively, this conclusion is somehow against the common sense that a larger frequency of disease-related genotype would bring more interaction effect. However, consider a dataset with all samples having the same genotype. Then there are no occurrences of other genotypes and, thus, this interaction model behaves like a baseline disease rate, which cannot then be detected as a (separate) interaction.**

**Next, we consider the case in our simulation study**. As explained in the main text, in our simulation, we adjust the sporadic rate so that the number of cases and the number of controls are approximately equal in each dataset. Under this constraint, we investigate the relationship between P value, frequency of the disease-related genotypes$B$, and the penetrance value $m$. From Table 2, we can write the constraint as:

$$\begin{matrix} k+NBp+N(1-B)q-k=N\eta\\ \Rightarrow Bp+(1-B)q=\eta\end{matrix}$$

Since $p=m+(1-m)\cdot q$, we have

$q=\frac{\eta-Bm}{1-Bm}$ (5)

where $\eta\approx0.5$ is the proportion of cases in each dataset. Substituting (5) in (3), we have

$\begin{matrix} x_{0}=\frac{NBp-NB[Bp+(1-B)q]}{\sqrt{NB(1-B)[Bp+(1-B)q][B(1-p)+(1-B)(1-q)]}} \\ =\frac{NB\cdot(p-\eta)}{\sqrt{NB(1-B)\eta(1-\eta)}} \\ =\frac{\sqrt{N}}{\sqrt{\eta(1-\eta)}}\cdot(p-\eta)\cdot\sqrt{\frac{B}{1-B}} ∵p=m+(1-m)\cdot q, q=\frac{\eta-Bm}{1-Bm} \\ =\frac{\sqrt{N}}{\sqrt{\eta(1-\eta)}}\cdot\frac{m(1-B)(1-\eta)}{1-Bm}\cdot\sqrt{\frac{B}{1-B}} \end{matrix}$ (6)

In Equation (6), $N$ and $\eta$ are fixed and considered as constants. When $B$ is fixed, (6) becomes $x_{0}=\frac{\sqrt{N}\cdot(1-\eta)}{\sqrt{\eta(1-\eta)}}\cdot\sqrt{(1-B)B}\frac{m}{1-Bm}$. As $m$ becomes larger, $x_{0}$ will become larger, and the P value of the interaction model $\text{erfc}(x_{0})$ will become smaller. When $m$ is fixed, from (6):

$$\begin{matrix} x_{0}=m\sqrt{N}\cdot\sqrt{\frac{(1-\eta)}{\eta}}\cdot\sqrt{\frac{(1-B)B}{(1-Bm)^{2}}} \\ =m\sqrt{N}\cdot\sqrt{\frac{(1-\eta)}{\eta}}\cdot\sqrt{y} \end{matrix}$$

where $y=\frac{(1-B)B}{(1-Bm)^{2}}$. $x_{0}$ is increasing for increasing $y$. Thus we have

$$\begin{matrix} \frac{dy}{dB}=\frac{(1-2B)(1-mB)^{2}-(1-B)\cdot B\cdot2(1-mB)\cdot(-m)}{\left(1-mB\right)^{4}} \\ =\frac{(1-2B)(1-mB)-(1-B)\cdot B\cdot2\cdot(-m)}{\left(1-mB\right)^{3}} \\ =\frac{1-(2-m)B}{\left(1-mB\right)^{3}}. \end{matrix}$$

Since$1-mB>0$, when $B<\frac{1}{2-m}$, $\frac{dy}{dB}>0$; otherwise $\frac{dy}{dB}\leq0$. Thus, when $B<\frac{1}{2-m}$, as $B$becomes larger, $y$ will become larger,$x_{0}$ will become larger, and the P value of the interaction model $\text{erfc}(x_{0})$ will become smaller; when $B>\frac{1}{2-m}$, as $B$becomes larger, the P value will become larger. So we have the following results:

***Conclusion 1:***

***In our simulation study (with the ratio between the number of cases and that of controls being fixed), the P value of a interaction model is decreasing for increasing penetrance value $m$. Moreover, the P value of a interaction model is decreasing for increasing $B$ when $B<\frac{1}{2-m}$, and increasing for increasing $B$when $B>\frac{1}{2-m}$.***

This result is consistent with common prior knowledge in genetic association testing. **It is also consistent with our observations in Figs. 2, 3, 4, 5** -- with larger penetrance multiplier parameter θ, more detection power (definition 1) is obtained by the methods for all the interaction models. Since the disease-related genotype frequency of models 2, 3, 4, 5 is quite small ($B<\frac{1}{2-m}$), with larger MAF multiplier parameter β, there is a larger frequency $B$of disease-related genotypes for models 2, 3, 4, 5. Again consistent with our theoretical conclusions, more detection power (definition 1) overall is experimentally observed to be obtained by the methods on interaction models 2, 3, 4, 5 (see Figs. 2, 3, 4, 5).

However, for model 1, whose disease-related genotypes are formed by major alleles, the situation is different. The disease-related genotype frequency $B$ is large ($B>\frac{1}{2-m}$), so from theoretical conclusion 1, the statistical significance is expected to be decreasing for increasing $B$. Also, different from models 2, 3, 4, 5, in model 1$B$is decreasing for increasing MAF. Therefore, theoretically, the significance of the interaction effect should be larger as the MAF increases. **By looking at Fig. 1, we observe that the powers (definition 1) of the methods do increase when $\beta$ is larger, which coincides with our theoretical result.**

**However, we also note that conclusion 1 is based on the ideal case, where it is assumed all ground-truth interactions are evaluated by the heuristic search and estimated accurately. Actually, *main* effects of the ground-truth interactions may also enhance power (under definition 1)**. So we need to step back from the conclusion that the joint effects fully explain the increased power when we increase θ and β. Note that Fig. 9 in section 4.2 demonstrates an important problem with these detection methods: they fail to detect most interaction effects, but rather detect interacting SNPs by their main effects. By comparing Fig. 1 and Fig. 9, we see that models 1, 3, 4, and 5 are rarely detected jointly as interactions; instead, they are (partly) detected by their main effects. Therefore the significance of the interaction is not sufficient to explain the power changes in these models. We also still need to further analyze how the main effect of an interaction model changes when we adjust the penetrance of the interaction model and the MAF.

*1.1.C. The relationship between main effect of an interaction model, penetrance value, and the frequency of disease-related genotypes*

For simplicity, let us first look at a 2-way interaction model with dominant or recessive allele coding. Similar to (1), we assume that the disease-related genotypes have uniform penetrance value. So we can shrink the 3x3 penetrance table into a 2x2 table as in Table 3, where the disease-related genotype is the shaded grid. For the disease-related genotype, let its frequency be $a\cdot b$, where $a$ is from SNP A and $b$ is from SNP B. Similar to (2), let $q$ denote the baseline disease rate and $m$ denote the penetrance value of the disease-related genotypes. We want to determine the relationship among $a, b, m$ and the main effects of SNP A and SNP B.

**Table 3.** Penetrance table for a simplified 2-way interaction model. The part with the dashed line is not the penetrance table, but rather indicates the marginal frequencies for SNP A and SNP B.

| $0$ | $0$ | 1-a |
| --- | --- | --- |
| $0$ | $m$ | a |
| 1-b | b |  |

By adding the baseline disease rate, Table 3 becomes

| $q$ | $q$ | 1-a |
| --- | --- | --- |
| $q$ | $p$ | a |
| 1-b | b |  |

, where $p=m+(1-m)q$. By projecting this interaction model onto SNP A, we have the main penetrance table of A as:

**Table 4.** Main penetrance table of SNP A. The part with the dashed line is not the penetrance table, but rather the genotype frequencies.

| $bp+(1-b)q$ | $q$ |
| --- | --- |
| $a$ | $1-a$ |

Let $\mathbf{G}_{A}$ be the genotypes with frequency $a$. From Table 4, we construct the contingency table as

**Table 5.** Contingency table for the main effect of SNP A

|  | $g\in\mathbf{G}_{A}$ | $g\notin\mathbf{G}_{A}$ |
| --- | --- | --- |
| Disease | $Na(bp+(1-b)q)$ | $N(1-a)q$ |
| Normal | $Na(1-bp-(1-b)q)$ | $N(1-a)(1-q)$ |

Similar to section 1.1.A, we define the number of cases carrying $g\in\mathbf{G}_{A}$ as the random variable $K$. $K$ follows the hypergeometric distribution with mean

$$\text{E}[K]=\frac{N(abp-abq+q)Na}{N}=Na(abp-abq+q)$$

and variance

$\begin{matrix} \text{Var}\left(K\right)=\frac{N(abp-abq+q)N(1-abp+abq-q)N(1-a)Na}{N^{2}(N-1)} \\ \approx N(abp-abq+q)(1-abp+abq-q)(1-a)a \end{matrix}$.

Then we asymptotically invoke the Gaussian distribution to calculate the P value as:

$\begin{matrix} \text{erfc}\left(\frac{Na(bp+(1-b)q)-Na(abp-abq+q)}{\sqrt{N(abp-abq+q)(1-abp+abq-q)(1-a)a}}\right) \\ \text{=erfc}\left(\frac{Nab(p-q)(1-a)}{\sqrt{N(abp-abq+q)(1-abp+abq-q)(1-a)a}}\right) \\ \text{=erfc}\left(\sqrt{Na(1-a)}\cdot\frac{b(p-q)}{\sqrt{(abp-abq+q)(1-abp+abq-q)}}\right). \end{matrix}$ (7)

Equation (7) is quite complicated and not necessarily monotonic. But in our simulation study, we add the constraint that the proportion of cases in each dataset ($\eta$) is fixed, so from Table 5 we have:

$\begin{matrix} Na(bp+(1-b)q)+N(1-a)q=N\eta\\ \Rightarrow abp-abq+q=\eta\end{matrix}$ (8)

Substituting (8) in (7), we obtain the P value as

$\begin{matrix} \text{erfc}\left(\sqrt{Na(1-a)}\cdot\frac{b(p-q)}{\sqrt{(abp-abq+q)(1-abp+abq-q)}}\right) \\ =\text{erfc}\left(\sqrt{Na(1-a)}\cdot\frac{b(p-q)}{\sqrt{\eta(1-\eta)}}\right) ∵abp-abq+q=\eta\\ =\text{erfc}\left(\sqrt{Na(1-a)}\cdot\frac{bm}{\sqrt{\eta(1-\eta)}}(1-q)\right) ∵q=\frac{\eta-abm}{1-abm} from (5) \\ =\text{erfc}\left(\sqrt{Na(1-a)}\cdot\frac{1}{\frac{1}{bm}-a}\sqrt{\frac{1-\eta}{\eta}}\right) \end{matrix}$ (9)

, where $N$ and $\eta$ are constants,

From (9), we obtain that the P value is decreasing for increasing $b$; and the P value is decreasing for increasing $m$. Also let $z=\frac{a-a^{2}}{(1-bma)^{2}}$, the P value becomes:

$$\text{erfc}\left(\sqrt{N}\cdot\sqrt{z}\cdot bm\cdot\sqrt{\frac{1-\eta}{\eta}}\right)$$

, which is decreasing for increasing $z$. Then we have

$$\begin{matrix} \frac{dz}{da}=\frac{(1-bma)^{2}(1-2a)-2(a-a^{2})(1-bma)(-bm)}{(1-bma)^{4}} \\ =\frac{(1-bma)(1-2a)-2(a-a^{2})(-bm)}{(1-bma)^{3}} \\ =\frac{1-(2-bm)a}{(1-bma)^{3}}. \end{matrix}$$

So when $a<\frac{1}{2-bm}$, $z$ is increasing for increasing $a$, thus the P value is decreasing for increasing $a$; when $a>\frac{1}{2-bm}$, $z$ is decreasing for increasing $a$, thus the P value is increasing for increasing $a$.

***Conclusion 2: In our simulation study (wherein the ratio between the number of cases and controls being fixed), for the interaction between SNP A and SNP B, the P value for the main effects of SNP A is decreasing for increasing penetrance value $m$at the disease-related genotype. The P value for the main effects of SNP A is decreasing for increasing marginal frequency $b$ of other SNP in this interaction model. When $a\in(0, \frac{1}{2-bm}]$, the P value is decreasing for increasing marginal frequency $a$ of the disease-related genotype. But when $a\in(\frac{1}{2-bm}, 1)$, the P value is increasing for increasing marginal frequency $a$ of the disease-related genotype.***

**In most cases,** not all of $a$, $b$, and $m$ will be large simultaneously (otherwise, the disease would be prevailing). So we can assume their product$abm<<1$, and (9) can be rewritten as

$\text{erfc}\left(\sqrt{Na(1-a)}\cdot\frac{1}{\frac{1}{bm}-a}\sqrt{\frac{1-\eta}{\eta}}\right)\approx\text{erfc}\left(\sqrt{Na(1-a)}\cdot bm\cdot\sqrt{\frac{1-\eta}{\eta}}\right)$

**, and the P value is increasing for increasing** *a* ***when* $a\in(0.5, 1)$.**

Similar conclusions can be obtained for higher-order interactions.

**1.2 The theoretical conclusions well support the comparison results**

The theoretical conclusions in section 1.1 well explain our experimental comparison results. From Fig. 9, we observed that **models 1, 3, 4, and 5** **are rarely detected as full interactions** by the methods; instead, their main effects are detected (see Fig. 1, Fig. 9); also, some methods can detect the interaction effects of model 2. Accordingly, **the theoretical analysis of main effects is consistent with and thus reasonably explains the experimentally observed relationship between power, MAF, and penetrance for models 1, 3, 4 and 5**. Likewise, the theoretical analyses of **both main and interaction effects reasonably explain the observed relationship between power, MAF, and penetrance for model 2**.

*1.2.A. Power (definition 1) goes down when we decrease $\beta$ or $\theta$.*

For models 3, 4, and 5, of which the power is mainly decided by main effect, the disease-related genotypes are associated with minor alleles, so a larger MAF multiplier parameter$\beta$ will lead to larger $a$ and $b$. Also, it is easy to see that the main frequencies of disease genotypes are quite small. Based on ***Conclusion 2*,** the P value is decreasing for increasing $a$ (when $a$ is small), $b$, and $m$. As $a$ and $b$ are increasing for increasing$\beta$, and $m$ is increasing for increasing $\theta$, the P value is decreasing for increasing $\beta$ and $\theta$, **indicating that a smaller $\beta$ or $\theta$ means weaker main effect.** For model 2, of which power is mainly decided by interaction effect, ***Conclusion 1 also indicates a smaller*$\beta$ or $\theta$ means weaker main effect**. **So for models 2, 3, 4, 5, it is reasonable to observe that the power (definition 1) goes down when we decrease $\beta$ or$\theta$, as shown in Fig. 2, 3, 4, 5.**

For model 1, the disease-related genotypes are associated with major alleles. So we use the following analysis to further explore the relationship between power, $\beta$ and $\theta$. The penetrance table of Model A can be rewritten as:

|  | $(1-0.25\beta)^{2}$ | $2(1-0.25\beta)0.25\beta$ | $\left(0.25\beta\right)^{2}$ |
| --- | --- | --- | --- |
| $(1-0.25\beta)^{2}$ | $0.07\theta$ | $0.07\theta$ | 0 |
| $2(1-0.25\beta)0.25\beta$ | $0.07\theta$ | $0.07\theta$ | 0 |
| $(0.25\beta)^{2}$ | 0 | 0 | 0 |

, where the first row and first column denote the frequency of genotypes. The MAFs for both SNP A and B are $0.25\beta$. By combining the disease-related genotypes, we get

|  | $1-(0.25\beta)^{2}$ | $(0.25\beta)^{2}$ |
| --- | --- | --- |
| $1-(0.25\beta)^{2}$ | $0.07\theta$ | 0 |
| $(0.25\beta)^{2}$ | 0 | 0 |

Corresponding to Table 3, we have$a=b=1-(0.25\beta)^{2}$, $m=0.07\theta$. Since$m=0.07\theta<<1, a<1, b<1$, we have$abm<<1$. The P value in equation (9) can then be written as:

$$\begin{matrix} \text{erfc}\left(\sqrt{Na(1-a)}\cdot\frac{1}{\frac{1}{bm}-a}\sqrt{\frac{1-\eta}{\eta}}\right) ∵a=b, abm<<1 \\ \approx\text{erfc}\left(\sqrt{Na^{3}(1-a)}\cdot m\cdot\sqrt{\frac{1-\eta}{\eta}}\right) \end{matrix}$$

Because $\beta\in(0,1)$, $a=1-(0.25\beta)^{2}$, $a\in(0.9375,1)$. Thus, it is easy to see that $a^{3}(1-a)$ is decreasing for increasing $a$, and so the P value is increasing for increasing $a$**. Since a larger $\beta$ means smaller $a$ and $b$, we infer that the P value is decreasing for increasing $\beta$, i.e., a larger $\beta$ means stronger main effects**. **Therefore, it is reasonable that that all the methods have higher power for model 1 when $\beta$ is increasing, as shown in Fig. 1.**

*1.2.B. The relationship between MAF and main effects of SNPs within an interaction model*

**An interesting finding in Fig. 11 is that while the MAF of SNP A is smaller than that of SNP B in model 2, SNP A is more likely to be detected by the methods. This phenomenon seems against our common sense, but note that we keep the proportion of cases fixed in each dataset by adjusting baseline disease rate (sporadic rate), and under this constraint, this phenomenon can be well explained by further analysis of equation (9).**

From penetrance of model 2, it is easy to infer that the marginal frequencies of the disease-related genotype ($a$ for SNP A and $b$ for SNP B) have relationship $b>a$. From (9), the P values for the main effects of A and B are

$$\text{PA=erfc}\left(\sqrt{Na(1-a)}\cdot\frac{bm(1-q)}{\sqrt{\eta(1-\eta)}}\right)$$

$$\text{PB=erfc}\left(\sqrt{Nb(1-b)}\cdot\frac{am(1-q)}{\sqrt{\eta(1-\eta)}}\right)$$

, respectively. Because

$$\begin{matrix} \sqrt{Na(1-a)}\cdot\frac{bm(1-q)}{\sqrt{\eta(1-\eta)}}-\sqrt{Nb(1-b)}\cdot\frac{am(1-q)}{\sqrt{\eta(1-\eta)}} \\ =\sqrt{\frac{N}{\eta(1-\eta)}}m(1-q)\left(\sqrt{ab^{2}-a^{2}b^{2}}-\sqrt{a^{2}b-a^{2}b^{2}}\right) ∵b>a, ab^{2}-a^{2}b^{2}>a^{2}b-a^{2}b^{2} \\ >0 \end{matrix}$$

, we have

$$\sqrt{Na(1-a)}\cdot\frac{bm(1-q)}{\sqrt{\eta(1-\eta)}}>\sqrt{Nb(1-b)}\cdot\frac{am(1-q)}{\sqrt{\eta(1-\eta)}}$$

, so

$\text{erfc}\left(\sqrt{Na(1-a)}\cdot\frac{bm(1-q)}{\sqrt{\eta(1-\eta)}}\right)<\text{erfc}\left(\sqrt{Nb(1-b)}\cdot\frac{am(1-q)}{\sqrt{\eta(1-\eta)}}\right)$, i.e.,$PA<PB$.

Therefore, the main effect of A are stronger than that of B. Thus it is reasonable to observe that the power for SNP A in Fig. 11(c) is greater than that for SNP B in Fig. 11(d).

**1.3. A summary of the theoretical work.**

Under the constraint that the proportion of cases is fixed in each dataset, the impacts of penetrance to both joint effect and main effect of an interaction model are quite direct: the larger penetrance value results in stronger joint effects of this interaction and stronger main effects of its SNP members.

Under the same constraint, the impact of the frequency of disease-related genotypes to the joint effect of an interaction model is also clear: joint effect is increasing for increasing frequency of disease-related genotypes if the frequency of disease-related genotypes is small ($B<\frac{1}{2-m}$), while decreasing for increasing frequency of disease-related genotypes otherwise. While the impact of the frequency of disease-related genotypes to the main effect of SNPs is more complicated. For the interaction between SNP A and SNP B, the P value for the main effects of SNP A is decreasing for increasing penetrance value $m$at the disease-related genotype. The P value for the main effects of SNP A is decreasing for increasing marginal frequency $b$ of other SNP in this interaction model. When $a\in(0, \frac{1}{2-bm}]$, the P value is decreasing for increasing marginal frequency $a$ of the disease-related genotype. But when $a\in(\frac{1}{2-bm}, 1)$, the P value is increasing for increasing marginal frequency $a$ of the disease-related genotype.

**S2. Comprehensive power evaluation results (Power is defined as in the main text, i.e. definition 1)**

**Fig. 1.** The power of the 7 methods for basic model 1 with different parameter settings. Blue curve - SH, red curve - BEAM, magenta curve - FIM, green curve - MDR, black curve – IG, cyan curve – MECPM, yellow curve: LR.


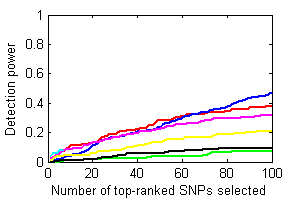

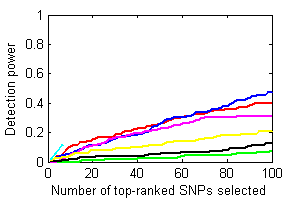

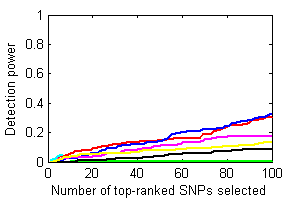


(a) *θ*=1.4, *β*=1, l=null (b) *θ*=1.4, *β*=0.9, l=null (c) *θ*=1.4, *β*=0.7, *l*=null


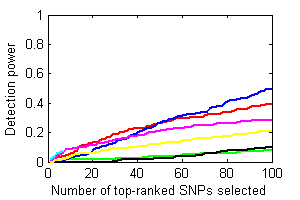

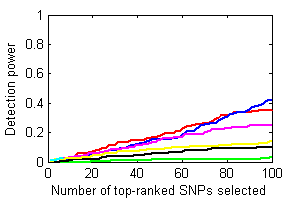

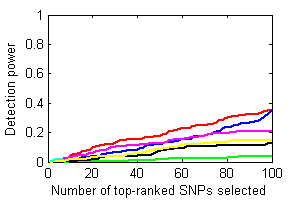


(d) *θ*=1.3, *β*=1, *l*=null (e) *θ*=1.3, *β*=0.9, *l*=null (f) *θ*=1.3, *β*=0.7, *l*=null


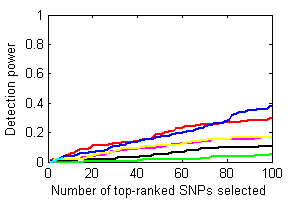

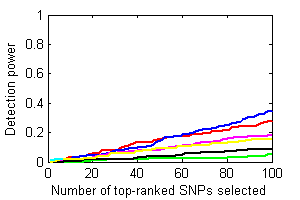

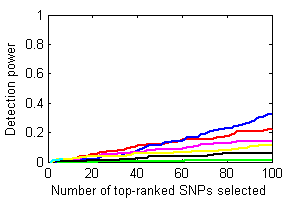


(g) *θ*=1, *β*=1, *l*=null (h) *θ*=1, *β*=0.9, *l*=null (i) *θ*=1, *β*=0.7, *l*=null


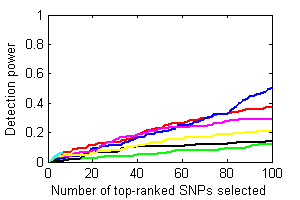

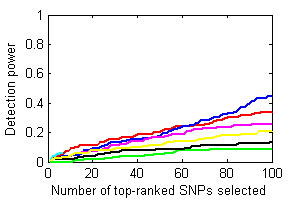

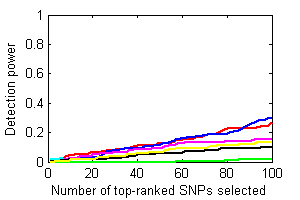


(j) *θ*=1.4, *β*=1, l=0.8 (k) *θ*=1.4, *β*=0.9, l=0.8 (l) *θ*=1.4, *β*=0.7, *l*=0.8


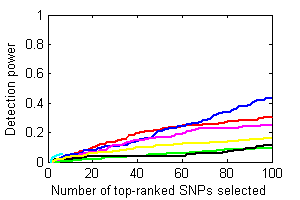

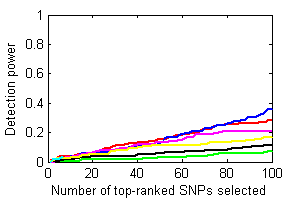

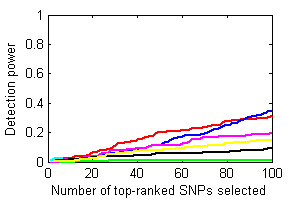


(m) *θ*=1.3, *β*=1, *l*=0.8 (n) *θ*=1.3, *β*=0.9, *l*=0.8 (o) *θ*=1.3, *β*=0.7, *l*=0.8


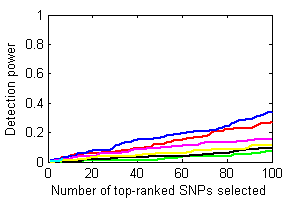

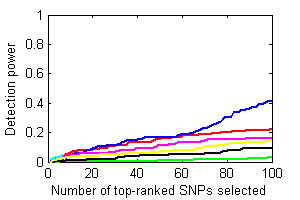

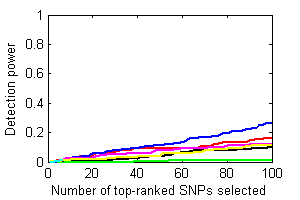


(p) *θ*=1, *β*=1, *l*=0.8 (q) *θ*=1, *β*=0.9, *l*=0.8 (r) *θ*=1, *β*=0.7, *l*=0.8

**Fig. 2.** The power of the 7 methods for basic model 2 with different parameter settings. Blue curve - SH, red curve - BEAM, magenta curve - FIM, green curve - MDR, black curve – IG, cyan curve – MECPM, yellow curve: LR.


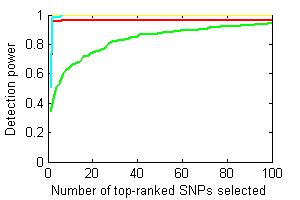

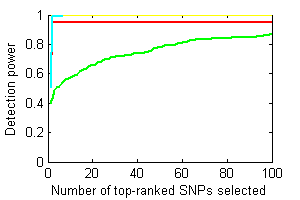

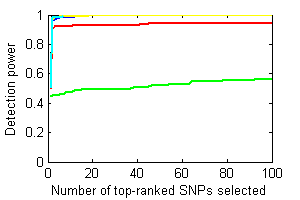


(a) *θ*=1.4, *β*=1, l=null (b) *θ*=1.4, *β*=0.9, l=null (c) *θ*=1.4, *β*=0.7, *l*=null


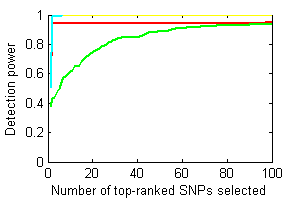

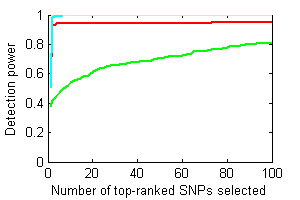

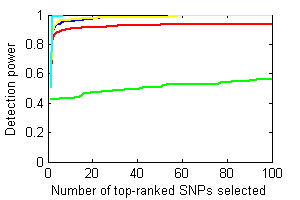


(d) *θ*=1.3, *β*=1, *l*=null (e) *θ*=1.3, *β*=0.9, *l*=null (f) *θ*=1.3, *β*=0.7, *l*=null


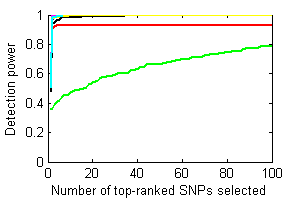

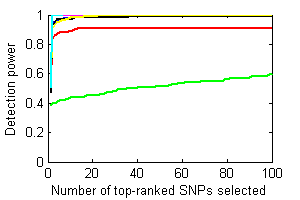

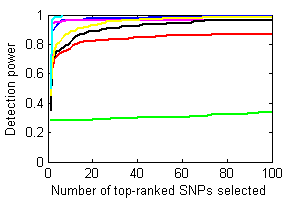


(g) *θ*=1, *β*=1, *l*=null (h) *θ*=1, *β*=0.9, *l*=null (i) *θ*=1, *β*=0.7, *l*=null


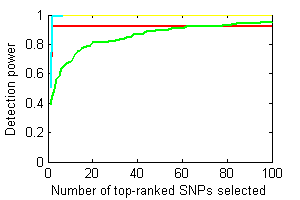

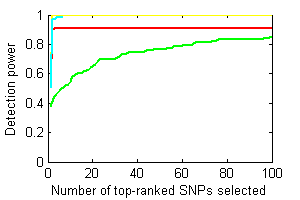

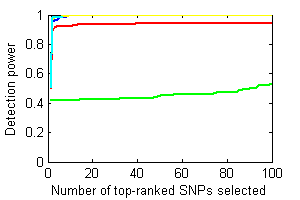


(j) *θ*=1.4, *β*=1, l=0.8 (k) *θ*=1.4, *β*=0.9, l=0.8 (l) *θ*=1.4, *β*=0.7, *l*=0.8


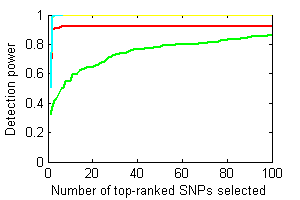

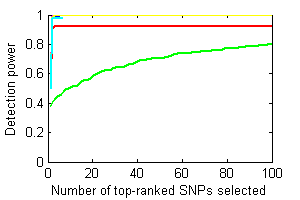

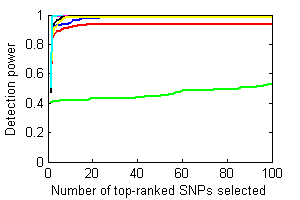


(m) *θ*=1.3, *β*=1, *l*=0.8 (n) *θ*=1.3, *β*=0.9, *l*=0.8 (o) *θ*=1.3, *β*=0.7, *l*=0.8


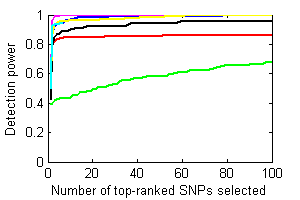

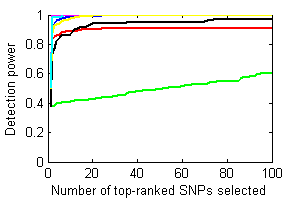

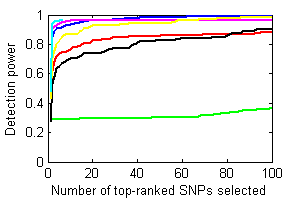


(p) *θ*=1, *β*=1, *l*=0.8 (q) *θ*=1, *β*=0.9, *l*=0.8 (r) *θ*=1, *β*=0.7, *l*=0.8

**Fig. 3.** The power of the 7 methods for basic model 3 with different parameter settings. Blue curve - SH, red curve - BEAM, magenta curve - FIM, green curve - MDR, black curve – IG, cyan curve – MECPM, yellow curve: LR.


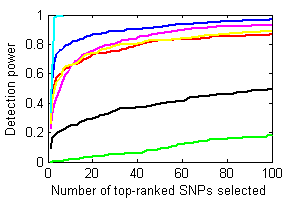

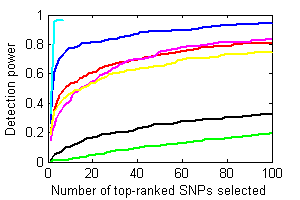

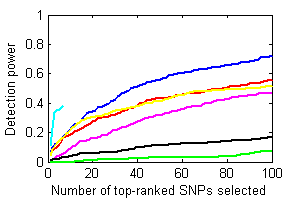


(a) *θ*=1.4, *β*=1, l=null (b) *θ*=1.4, *β*=0.9, l=null (c) *θ*=1.4, *β*=0.7, *l*=null


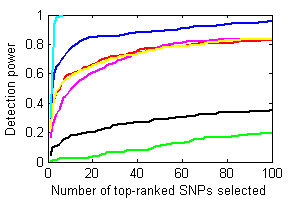

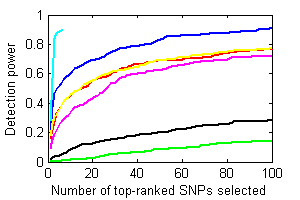

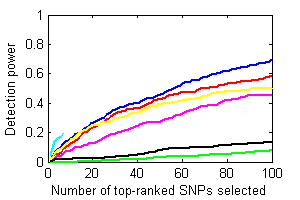


(d) *θ*=1.3, *β*=1, *l*=null (e) *θ*=1.3, *β*=0.9, *l*=null (f) *θ*=1.3, *β*=0.7, *l*=null


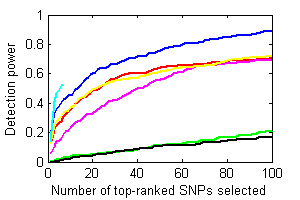

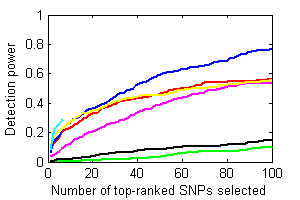

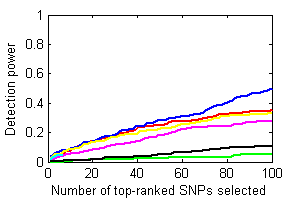


(g) *θ*=1, *β*=1, *l*=null (h) *θ*=1, *β*=0.9, *l*=null (i) *θ*=1, *β*=0.7, *l*=null


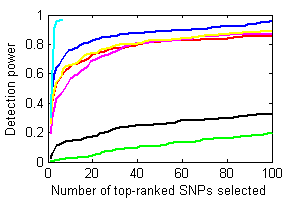

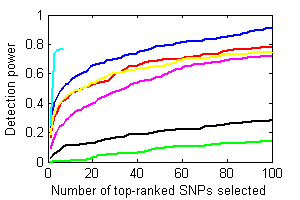

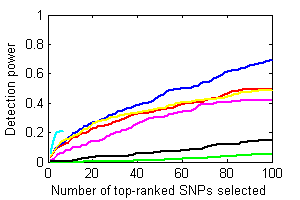


(j) *θ*=1.4, *β*=1, l=0.8 (k) *θ*=1.4, *β*=0.9, l=0.8 (l) *θ*=1.4, *β*=0.7, *l*=0.8


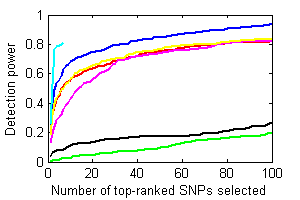

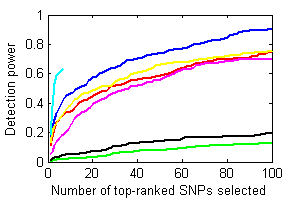

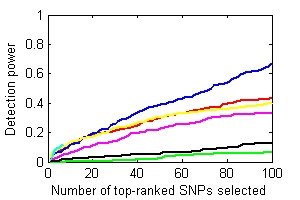


(m) *θ*=1.3, *β*=1, *l*=0.8 (n) *θ*=1.3, *β*=0.9, *l*=0.8 (o) *θ*=1.3, *β*=0.7, *l*=0.8


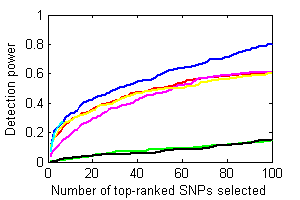

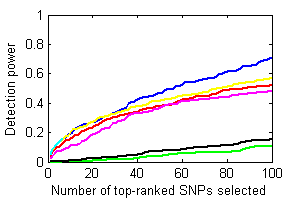

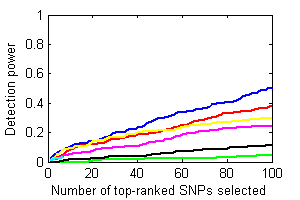


(p) *θ*=1, *β*=1, *l*=0.8 (q) *θ*=1, *β*=0.9, *l*=0.8 (r) *θ*=1, *β*=0.7, *l*=0.8

**Fig. 4.** The power of the 7 methods for basic model 4 with different parameter settings. Blue curve - SH, red curve - BEAM, magenta curve - FIM, green curve - MDR, black curve – IG, cyan curve – MECPM, yellow curve: LR.


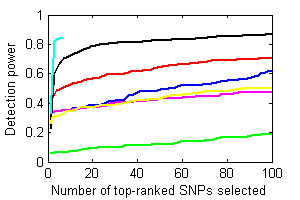

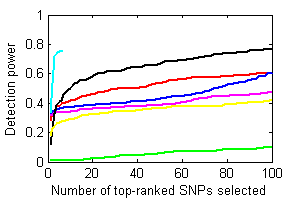

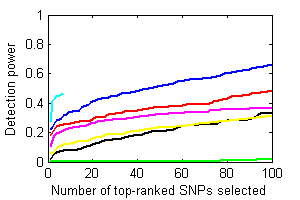


(a) *θ*=1.4, *β*=1, l=null (b) *θ*=1.4, *β*=0.9, l=null (c) *θ*=1.4, *β*=0.7, *l*=null


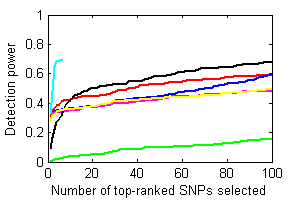

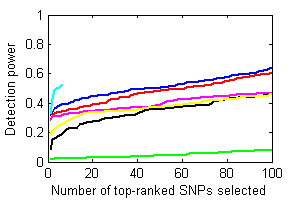

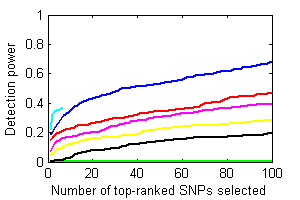


(d) *θ*=1.3, *β*=1, *l*=null (e) *θ*=1.3, *β*=0.9, *l*=null (f) *θ*=1.3, *β*=0.7, *l*=null


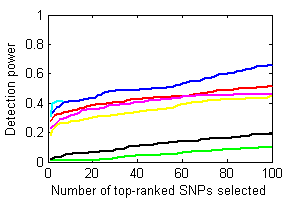

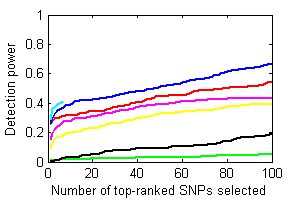

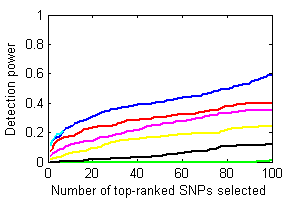


(g) *θ*=1, *β*=1, *l*=null (h) *θ*=1, *β*=0.9, *l*=null (i) *θ*=1, *β*=0.7, *l*=null


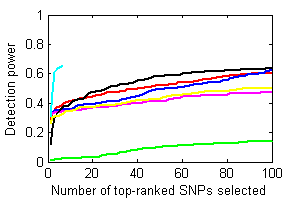

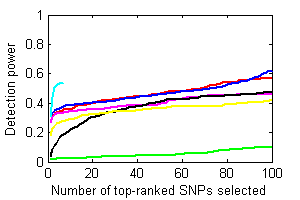

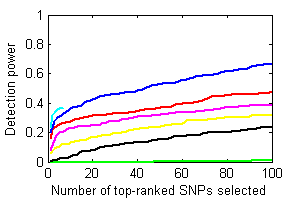


(j) *θ*=1.4, *β*=1, l=0.8 (k) *θ*=1.4, *β*=0.9, l=0.8 (l) *θ*=1.4, *β*=0.7, *l*=0.8


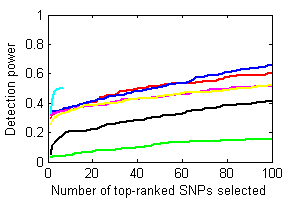

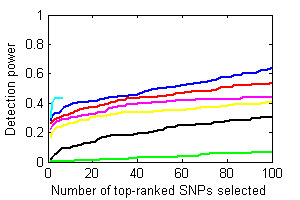


(m) *θ*=1.3, *β*=1, *l*=0.8 (n) *θ*=1.3, *β*=0.9, *l*=0.8 (o) *θ*=1.3, *β*=0.7, *l*=0.8


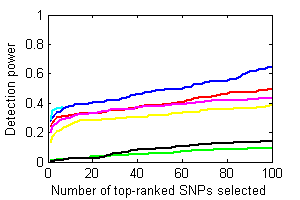

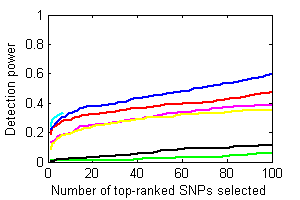

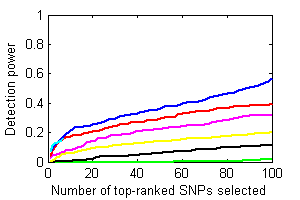


(p) *θ*=1, *β*=1, *l*=0.8 (q) *θ*=1, *β*=0.9, *l*=0.8 (r) *θ*=1, *β*=0.7, *l*=0.8

**Fig. 5.** The power of the 7 methods for basic model 5 with different parameter settings. Blue curve - SH, red curve - BEAM, magenta curve - FIM, green curve - MDR, black curve – IG, cyan curve – MECPM, yellow curve: LR.


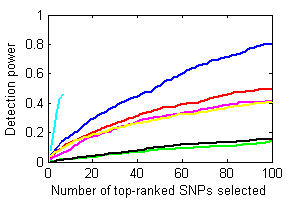

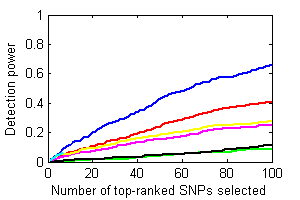

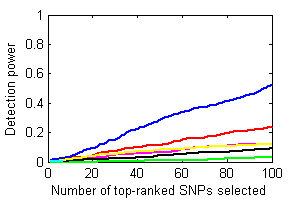


(a) *θ*=1.4, *β*=1, l=null (b) *θ*=1.4, *β*=0.9, l=null (c) *θ*=1.4, *β*=0.7, *l*=null


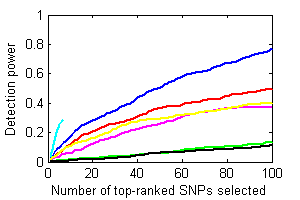

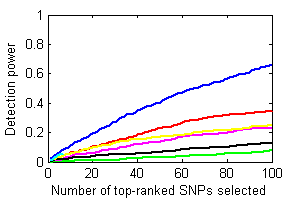

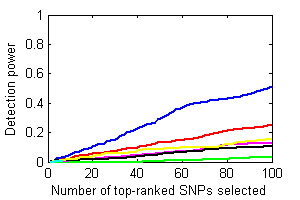


(d) *θ*=1.3, *β*=1, *l*=null (e) *θ*=1.3, *β*=0.9, *l*=null (f) *θ*=1.3, *β*=0.7, *l*=null


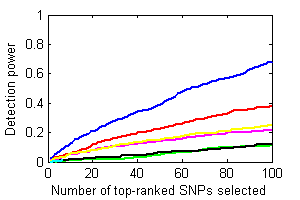

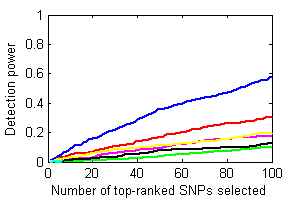

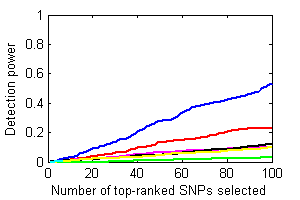


(g) *θ*=1, *β*=1, *l*=null (h) *θ*=1, *β*=0.9, *l*=null (i) *θ*=1, *β*=0.7, *l*=null


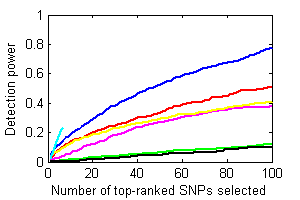

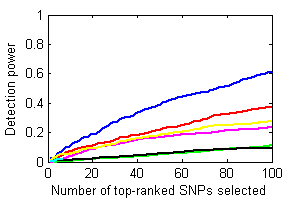

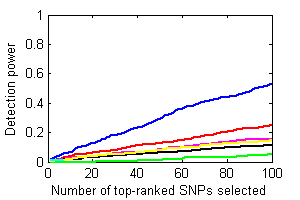


(j) *θ*=1.4, *β*=1, l=0.8 (k) *θ*=1.4, *β*=0.9, l=0.8 (l) *θ*=1.4, *β*=0.7, *l*=0.8


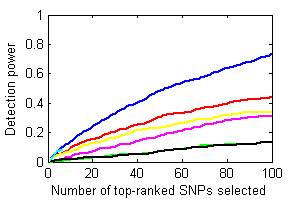

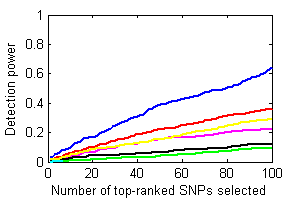

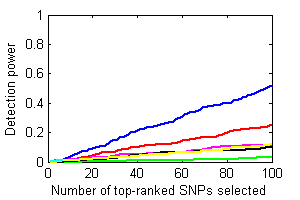


(m) *θ*=1.3, *β*=1, *l*=0.8 (n) *θ*=1.3, *β*=0.9, *l*=0.8 (o) *θ*=1.3, *β*=0.7, *l*=0.8


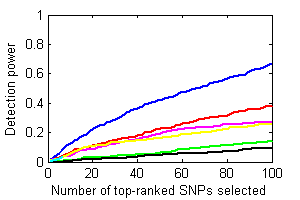

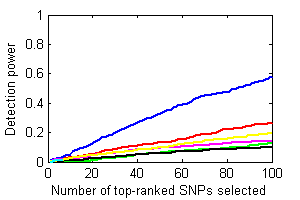

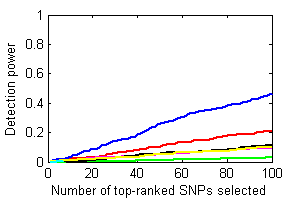


(p) *θ*=1, *β*=1, *l*=0.8 (q) *θ*=1, *β*=0.9, *l*=0.8 (r) *θ*=1, *β*=0.7, *l*=0.8

**Fig. 6.** The power of the 7 methods for the whole 15-ground-truth-SNP set with different parameter settings. Blue curve - SH, red curve - BEAM, magenta curve - FIM, green curve - MDR, black curve – IG, cyan curve – MECPM, yellow curve: LR.


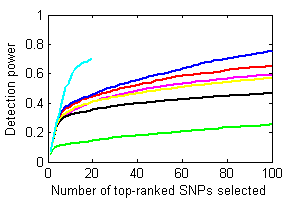

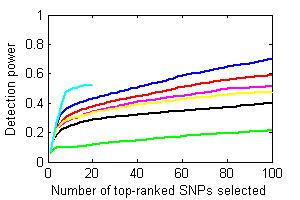

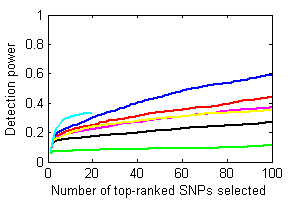


(a) *θ*=1.4, *β*=1, l=null (b) *θ*=1.4, *β*=0.9, l=null (c) *θ*=1.4, *β*=0.7, *l*=null


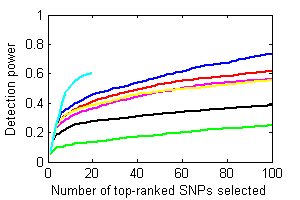

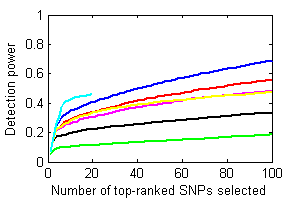

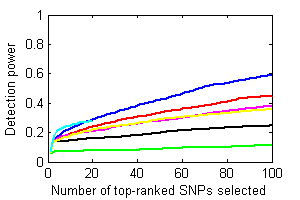


(d) *θ*=1.3, *β*=1, *l*=null (e) *θ*=1.3, *β*=0.9, *l*=null (f) *θ*=1.3, *β*=0.7, *l*=null


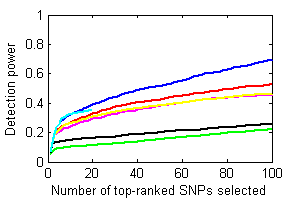

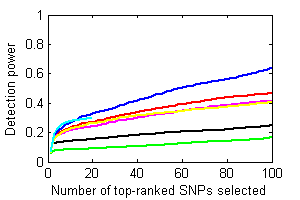

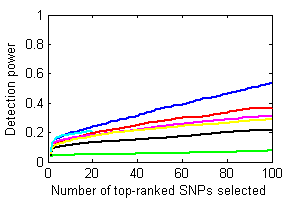


(g) *θ*=1, *β*=1, *l*=null (h) *θ*=1, *β*=0.9, *l*=null (i) *θ*=1, *β*=0.7, *l*=null


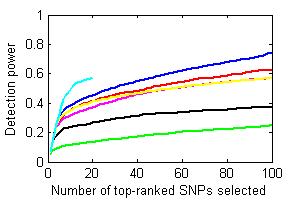

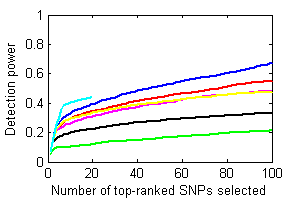


(j) *θ*=1.4, *β*=1, l=0.8 (k) *θ*=1.4, *β*=0.9, l=0.8 (l) *θ*=1.4, *β*=0.7, *l*=0.8

(m) *θ*=1.3, *β*=1, *l*=0.8 (n) *θ*=1.3, *β*=0.9, *l*=0.8 (o) *θ*=1.3, *β*=0.7, *l*=0.8

(p) *θ*=1, *β*=1, *l*=0.8 (q) *θ*=1, *β*=0.9, *l*=0.8 (r) *θ*=1, *β*=0.7, *l*=0.8

**Fig. 7.** The reproducibility of the 5 methods for the whole 15-ground-truth-SNP set with the parameter setting {θ=1.4, β=1, l=null). The height of the band around power curves shows the standard deviation of power. Blue curve - SH, red curve - BEAM, magenta curve - FIM, green curve - MDR, black curve – IG.

**Findings and explanations:**

In Fig. 1, 2, 3, 4, 5, 6, we observe that the power curves of most methods go up quickly with K when K is small, then increase more gradually for K above an inflection point (with K the number of top ranking SNPs). This behavior is next explained.

The abrupt power increase at small K is mainly associated with main effects of SNPs. The methods easily detect ground-truth SNPs with strong main effects (e.g., SNPs in models 2, 3, and 4) at the beginning, and then fail to differentiate ground-truth SNPs with weak main effects from null SNPs. Thus, most methods have a steep power curve when K is small; subsequently, the power curves increase slowly.

**S3. ROC curves for the methods**

**Fig. 8.** ROC curves for the whole ground-truth SNP set, under different parameter settings. The width of the band around each point on a curve is half the standard deviation of sensitivity on this point. Blue curve - SH, red curve - BEAM, magenta curve - FIM, green curve - MDR, black curve – IG.

(a) *θ*=1.4, *β*=1, l=null (b) *θ*=1.4, *β*=0.9, l=null (c) *θ*=1.4, *β*=0.7, *l*=null

(d) *θ*=1.3, *β*=1, *l*=null (e) *θ*=1.3, *β*=0.9, *l*=null (f) *θ*=1.3, *β*=0.7, *l*=null

(g) *θ*=1, *β*=1, *l*=null (h) *θ*=1, *β*=0.9, *l*=null (i) *θ*=1, *β*=0.7, *l*=null

(j) *θ*=1.4, *β*=1, l=0.8 (k) *θ*=1.4, *β*=0.9, l=0.8 (l) *θ*=1.4, *β*=0.7, *l*=0.8

(m) *θ*=1.3, *β*=1, *l*=0.8 (n) *θ*=1.3, *β*=0.9, *l*=0.8 (o) *θ*=1.3, *β*=0.7, *l*=0.8

(p) *θ*=1, *β*=1, *l*=0.8 (q) *θ*=1, *β*=0.9, *l*=0.8 (r) *θ*=1, *β*=0.7, *l*=0.8

**S4. Calculation of Effect Size for each interaction model**

We denote the effect size of an interaction model by the odds ratio between disease-related genotypes and disease-unrelated genotypes, i.e., we dichotomize the genotypes into a group with the lowest penetrance value (usually with “0” penetrance) and another group with higher penetrance values, and calculate the odds ratio between these two groups. In this way, for example, the $3\times3$ penetrance table for a two-way interaction is degraded to a $1\times2$ table.

Since in our simulation, we have multiple interaction models simultaneously existing in each dataset, we have to calculate the odds ratio by considering all the other interaction models present and the baseline sporadic rate.

Mathematically, let’s calculate odds ratio of model 1 as an example.

First we calculate the number of cases caused by each interaction model and the sporadic rate. M1’s penetrance can be simplified to

| M1 | $G_{11}^{A}$ or $G_{12}^{A}$ | $G_{22}^{A}$ |
| --- | --- | --- |
| $G_{11}^{B}$ or $G_{12}^{B}$ | 0.07 | 0 |
| $G_{22}^{B}$ | 0 | 0 |

So M1 is expected to make $R_{1}=0.07*P\left((g_{A}\in G_{11}^{A} or G_{12}^{A})\cap(g_{A}\in G_{11}^{B} or G_{12}^{B})\right)=0.0615$ of subjects get the disease. Similarly, M2 is expected to make $R_{2}=0.1016$ of subjects get the disease, M3 is expected to make $R_{3}=0.0464$ of subjects get the disease, M4 is expected to make $R_{4}=0.\text{0258 }$ of subjects get the disease, and M5 is expected to make $R_{5}=0.0217$ of subjects get the disease. Note that we assume models M1, M2, M3, M4, M5 and the sporadic rate $R_{0}$ independently contribute to disease risk, *i.e.*, the overall proportion of subjects carrying disease is $1-(1-R_{1})(1-R_{2})(1-R_{3})(1-R_{4})(1-R_{5})(1-R_{0})$. Since we want to make in total 50% of subjects carry disease, we have to adjust the sporadic rate $R_{0}$, so that

$1-(1-R_{1})(1-R_{2})(1-R_{3})(1-R_{4})(1-R_{5})(1-R_{0})=0.5$.

From the equation above, we obtain $R_{0}=0.3475$.

Second, we calculate odds ratio of model M1. From the number of cases caused by each interaction model and the sporadic rate, we can calculate the baseline disease rate for M1 as

$P_{1,base}=1-(1-R_{2})(1-R_{3})(1-R_{4})(1-R_{5})(1-R_{0})\text{=0.4672}$.

By dichotomizing the M1 penetrance table as two grids,

| M1 | $(g_{A}\in G_{11}^{A} or G_{12}^{A})\cap(g_{A}\in G_{11}^{B} or G_{12}^{B})$ | otherwise |
| --- | --- | --- |
|  | 0.07 | 0 |

the proportion of cases in the subjects carrying $(g_{A}\in G_{11}^{A} or G_{12}^{A})\cap(g_{A}\in G_{11}^{B} or G_{12}^{B})$ is

$p_{1}=1-(1-0.07)*(1-P_{1,base})=0.5045$;

and the proportion of cases in the subjects carrying genotypes other than $(g_{A}\in G_{11}^{A} or G_{12}^{A})\cap(g_{A}\in G_{11}^{B} or G_{12}^{B})$ is

$q_{1}=P_{1,base}=0.4672$.

So the odds ratio for M1 is calculated as

$$odds_{M1}=\frac{p_{1}(1-q_{1})}{(1-p_{1})q_{1}}=1.16$$

The odds ratio for other interaction models and main effects of individual SNPs can be calculated similarly.

**S5. Conservativeness of the** $\chi^{2}$ **statistics applied by SH and FIM**

As stated in the main text, we wanted to assess how much conservativeness comes purely from the summary statistics. For this purpose, we simulated 5,000 replicated datasets, each including 2 “null” SNPs and 200 samples, and then applied SH and FIM with exhaustive search to detect 2-way interactions. For this experiment, no Bonferroni correction is needed. Also, by design, this experiment eliminates the effects of heuristic search and SNP dependencies (which may stem both from multiple testing and from linkage disequilibrium, as we discussed in the main text). Thus, this experiment focuses solely on the accuracy of the statistic itself.

If the summary statistics are accurate, then the false positive rate should be consistent with the significance threshold. Based on Tables 6 and 7, we can see that when the significance threshold is set smaller, the false positive rate becomes more conservative (*i.e.,* the degree of overestimation of the empirical false positive rate is increasing). This explains why in Table 1 of the main text that false positive rate at 1st order is more accurate than at 2nd and 3rd orders (2nd and 3rd orders require much smaller significance thresholds). Thus we conclude that the summary statistics used in SH and FIM contribute to this conservativeness.

**Table 6.** Consistency for the $\chi^{2}$statistic (obtained from Pearson’s $\chi^{2}$ test used in SH) between the significance threshold and the false positive rate.

| Significance threshold | False positive rate | false positive rate / Significance threshold |
| --- | --- | --- |
| 0.5 | 0.5042 | 1.0084 |
| 0.1 | 0.0892 | 0.8920 |
| 0.01 | 0.0056 | 0.5600 |
| 0.005 | 0.0022 | 0.4400 |

**Table 7.** Consistency for the $\chi^{2}$statistic (obtained from the likelihood ratio test used in FIM) between the significance threshold and the false positive rate.

| Significance threshold | False positive rate | false positive rate / Significance threshold |
| --- | --- | --- |
| 0.5 | 0.4162 | 0.8324 |
| 0.1 | 0.0682 | 0.6820 |
| 0.01 | 0.0064 | 0.6400 |
| 0.005 | 0.0034 | 0.6800 |

**S6. Comparison of detection power and the number of false positive SNPs (false positive SNP count) under the 0.05 significance threshold**

We have shown that the significance assessment of the methods cannot serve as a reliable criterion for SNP detection. Here we want to experimentally show readers how this lack of reliability affects the detection of interactive SNPs. To assess this, we measured the detection power (definition 1) and the number of false positive SNPs under a given significance threshold for BEAM, SH, and FIM. The experiment is run on 100 replicated datasets in step 2 (each containing the 15 ground-truth SNPs) {*θ*=1.4, *β*=1, *l*=null}. Table 8 shows the averaged detection power and the number of false positive SNPs obtained by each of the methods.

**Table 8.** The detection power (power definition 1) and the number of false positive SNPs selected under significance level 0.05. The detection power and the number of false positive SNPs are averaged over 100 replicated datasets, for the parameter settings {*θ*=1.4, *β*=1, *l*=null}. There are 15 ground-truth SNPs and 985 null SNPs in each dateset.

|  | BEAM | SH | FIM |
| --- | --- | --- | --- |
| Power | 3.48/15=0.232 | 4.58/15=0.305 | 9.04/15=0.603 |
| Average number of false positive SNPs | 0.11 | 0.16 | 98.73 |

From Table 8, we can see that BEAM and SH detect few ground-truth SNPs but the number of false positives is also quite small. By contrast, FIM obtains more ground-truth SNPs at the cost of a (much) larger number of false positives.

It appears that FIM’s greater propensity for detecting true positive SNPs is caused by its giving less consideration to differentiating main effects from interaction effects. FIM, detects ground-truth SNPs with selecting many false positive interactions that include both ground-truth and null SNPs, i.e., the false interactions are considered significant because of the strong main effects of the involved ground-truth SNPs. In contrast, SH and BEAM penalize main effects when considering SNP interactions, and they have less false positives than FIM does. But the insensitive detection criteria of SH and BEAM limit their ability to find ground-truth interactions (and, thus, ground-truth SNPs).

Note that FIM’s number of false positive SNPs may appear contradictory to our conclusion that all 3 of the methods are conservative in terms of false positive rate. However, this is in fact reasonable when we consider the fact that we defined the false positive rate in step 1, which involved data sets with no ground-truth SNPs. When ground-truth SNPs *are* present, FIM detects false interactions that include them, which results in a (relatively) large number of false positives.

This comparison shows that even for the same significance level threshold, the three methods differ in both the detection power and the number of false positive SNPs. Actually it was the very finding which motivated us to do the step 1 experiment.
